# Expression of vimentin, TPI and MAT2A in human dermal microvascular endothelial cells during angiogenesis *in vitro*

**DOI:** 10.1371/journal.pone.0266774

**Published:** 2022-04-28

**Authors:** Christina Herre, Arpenik Nshdejan, Robert Klopfleisch, Giuliano Mario Corte, Mahtab Bahramsoltani

**Affiliations:** 1 Department of Veterinary Medicine, Institute of Veterinary Anatomy, Freie Universität Berlin, Berlin, Germany; 2 Department of Veterinary Medicine, Institute of Veterinary Pathology, Freie Universität Berlin, Berlin, Germany; University of Edinburgh, UNITED KINGDOM

## Abstract

**Introduction:**

*In vitro* assays of angiogenesis face immense problems considering their reproducibility based on the inhomogeneous characters of endothelial cells (ECs). It is necessary to detect influencing factors, which affect the angiogenic potency of ECs.

**Objective:**

This study aimed to analyse expression profiles of vimentin (VIM), triosephosphate isomerase (TPI) and adenosylmethionine synthetase isoform type–2 (MAT2A) during the whole angiogenic cascade *in vitro*. Furthermore, the impact of knocking down vimentin (VIM) on angiogenesis *in vitro* was evaluated, while monitoring TPI and MAT2A expression.

**Methods:**

A long–term cultivation and angiogenic stimulation of human dermal microvascular ECs was performed. Cells were characterized via VEGFR–1 and VEGFR–2 expression and a shRNA–mediated knockdown of VIM was performed. The process of angiogenesis *in vitro* was quantified via morphological staging and mRNA–and protein–levels of all proteins were analysed.

**Results:**

While native cells ran through the angiogenic cascade chronologically, knockdown cells only entered beginning stages of angiogenesis and died eventually. Cell cultures showing a higher VEGFR–1 expression survived exclusively and displayed an upregulation of MAT2A and TPI expression. Native cells highly expressed VIM in early stages, MAT2A mainly in the beginning and TPI during the course of angiogenesis *in vitro*.

**Conclusion:**

VIM knockdown led to a deceleration of angiogenesis *in* vitro and knockdown cells displayed expressional changes in TPI and MAT2A. Cell populations with a higher number of stalk cells emerged as being more stable against manipulations and native expression profiles provided an indication of VIM and MAT2A being relevant predominantly in beginning stages and TPI during the whole angiogenic cascade *in vitro*.

## Introduction

Angiogenesis is described as the growth and remodelling of new blood vessels from pre–existing ones [1–3]. During sprouting angiogenesis *in vivo*, vascular basement membrane degrades and endothelial tip cells migrate towards an angiogenic stimulus such as vascular endothelial growth factor A (VEGF-A). VEGF-A initiates tip cell activation and migration via vascular endothelial growth factor receptor–2 (VEGFR–2), which is abundant on filopodia. Induced by the expression of vascular endothelial growth factor receptor–1 (VEGFR–1), endothelial stalk cells elongate the sprout via proliferation. An internal lumen is being built and endothelial phalanx cells synthesise a new basement membrane, resulting in a mature vessel [3, 4].

The ability to run through these processes is indispensable for many pathological events, like tumour growth, ischemia and cardio vascular diseases [3]. Current effort in the research field of angiogenesis is focusing on tissue engineering and wound healing [5–7]. According to the 3Rs principle of replacing, reducing and refining animal experiments of Russel and Burch [8] and with the aim to reduce time and cost, *in vitro* models of angiogenesis are used frequently. However, due to the absence of a gold standard for these assays and the inhomogeneous character of endothelial cells (ECs), *in vitro* assays still face immense problems considering their reproducibility [6, 9–12].

Most *in vitro* assays cover only single stages of angiogenesis, e.g. migration, proliferation or tube formation [9, 10, 13]. However, the capacity of ECs to get stimulated for specific assays does not necessarily represent their potential to undergo the whole angiogenic cascade. While establishing an all–in–one assay, that comprises all stages of angiogenesis, it was shown that several batches of capillary–derived primary cell cultures of human microvascular endothelial cells display differences in their angiogenic potency. Irrespective of distributor recommended conditions of growth stimulation and cultivation, not all cells were able to undergo all stages of angiogenesis, resulting in the classification of ECs into angiogenic and non–angiogenic [14–17]. In order to investigate molecular differences and determine potential marker proteins, Bahramsoltani et al. [18] generated protein expression profiles of angiogenic and non–angiogenic ECs. By evaluating the expression patterns, seven proteins were found exclusively in angiogenic batches and only one protein in non–angiogenic. For this study, three proteins were determined to undergo further analysis.

Among the seven proteins found in angiogenic ECs, vimentin (VIM) is the most explored protein regarding its role in angiogenesis. The type III intermediate filament protein belongs to a protein family, which is mainly responsible for cell shape and motility. Therefore, VIM is shown to be expressed in all major human tissues, especially in highly proliferative and undifferentiated cells [19]. Besides its influence on the cell skeleton, VIM is described as a multifunctional protein that is involved in intracellular and extracellular signalling pathways and in mediating host cell invasion for viruses, including SARS–CoV, and different bacteria [20]. Considering angiogenesis, VIM is already established as a marker for immature angiogenic blood vessels [21]. By effecting the cytoskeleton and stabilizing the cell–matrix adhesion, it is highly involved in angiogenesis, especially during cell migration [19, 22] and sprouting [23, 24]. While passing the angiogenic cascade, it was shown that the expression of VIM in immature precursor cells decreased with maturation [10, 21]. Further studies showed the dynamic in VIM expression that may represent an adaptive mechanism of microvascular endothelial cells to stress. It may help cells to adjust their cell adhesion and motility to environmental conditions [25, 26]. Furthermore, knocking down models in mice led to insufficiencies in wound–healing, vessel remodelling and vasoactivity, without exposing the affected components and mechanisms. Up until today, the specific role of VIM in dermal angiogenesis is not fully revealed [19, 20, 23].

As a further protein, triosephosphate isomerase (TPI) was solely expressed in angiogenic ECs [18]. TPI is a glycolytic enzyme, which reaches its full catalytic activity by dimerization. It converts the isomers dihydroxyacetone phosphate into D–glyceraldehyde–3–phosphate reversely [27, 28]. Due to its involvement in glycolysis and gluconeogenesis, TPI establishes a connection to other metabolic mechanisms, i.e. lipid metabolism and pentose phosphate pathway. It represents an essential enzyme for cell metabolism which is highly present in all different kinds of human tissues [29]. It was demonstrated that stimulation of TPI induces an increase in ATP and leads to cell proliferation [28], a pivotal step in angiogenesis. In capillary ECs, it was shown that TPI expression was upregulated by induction of hypoxia [30]. Generally, there is hardly any information about TPI and its role in angiogenesis.

According to the proteomic approach of Bahramsoltani et al. [18], only one protein was found exclusively in non–angiogenic ECs, i.e. S–adenosylmethionine synthetase isoform type–2 (MAT2A). This enzyme is related to cell metabolism and is encoded by two genes, MAT1A–gene and MAT2A–gene. While MAT1A–gene is mostly expressed in liver tissue, MAT2A–gene is widely distributed in human tissues [31]. MAT2A is a highly conserved enzyme synthesising S–adenosylmethionine (SAM) by catalysing the reaction of methionine and adenosine triphosphate (ATP). SAM is a well–established and highly important compound, which serves as the major methyl donor in most methyl transfer reactions, including the methylation of proteins, nucleic acids and lipids [32]. Molecular methylation tends to have mainly regulatory functions, e.g. DNA–methylation results in the activation of cells to undergo maturation and differentiation [33]. Alternatively, hypomethylation of DNA promotes cells to migrate and proliferate. In ECs, the downregulation of DNA–methylation displayed an increase in VEGF–A expression resulting in a higher angiogenic potential [34]. In contrast, by increasing DNA–methylation due to implementation of SAM, ECs show a decreasing capacity to build capillary–like structures. This compound prevents ECs to undergo migration and proliferation [33, 34]. Until today, there is still rarely information about the enzyme S–adenosylmethionine synthetase isoform type–2 relating to ECs.

The aim of the present study was to determine the impact of knocking down VIM in human dermal microvascular endothelial cells (HDMECs) during the angiogenic cascade *in vitro*. Within this process, expressional changes in TPI and MAT2A mRNA and protein levels were analysed. Furthermore, the experimental setup was designed to exhibit time–dependant fluctuations in VIM, TPI and MAT2A expression during angiogenesis *in vitro*.

## Cells, materials and methods

### Plasmids, primers and shRNA

Initially, five constructs were designed for the expression of short hairpin RNA targeting VIM–mRNA (shVIM). The generation of shVIM was carried out as previously described for FoxP2 [35]. In brief, the linear DNA coding for the constructs of shVIM was structured sense–loop–antisense. The loop sequence was GTGAAGCCACAGATG. The knockdown efficiency of each hairpin construct was tested in HEK 293T cells *in vitro*. Concurrently, an overexpression of VIM, tagged with V5 epitope was induced (VIM^+^, shown in S1 Table). By using V5 antibodies (Abcam, Cambridge, UK, ab15828, 1:5,000) as primary and Rabbit IgG (GE Healthcare, Freiburg, Germany, NA9340, 1:10,000) as secondary antibodies, Western blot analysis determined the construct with the strongest reduction in VIM expression (shVIM target sequence GGCACGTCTTGACCTTGAACG). The DNA fragments were subcloned into the lentiviral expression vector pFUGW, that was modified by the addition of an U6 promotor with the aim to raise its expression efficacy [36]. For visual infection control, the modified vector contained the information for enhanced green fluorescent protein (eGFP). Additionally, a nontargeting hairpin (shSCR target sequence GAGAGCCGTCCCGGTCTATTA) was subcloned into the vector to be used as a control. The generation of lentivirus was implemented as previously published [36]. Viral titers were maintained in the range of 1–3 x 10^7^ IU/μl and viral particles were added to the cells in a 50–fold concentration.

### Cells, media and cultivation

Two batches of human microvascular endothelial cells derived from neonatal foreskin (HD1 and HD2) were acquired from LONZA Bioscience (Basel, Switzerland, HMVEC–dBl–Neo, Cat. No. CC–2813). Endothelial cell population was guaranteed by the distributor’s certified analysis of CD31/105 and von Willebrand Factor VIII expression and positive uptake for acetylated low density lipoprotein. EBM^TM^–2 Endothelial Cell Growth Basal Medium–2 (LONZA, Basel, Switzerland, Cat. No. CC–00190860) was used as a basal medium (BM). For facilitating cell survival and provoking an angiogenic response, EGM^TM^–2 MV Microvascular Endothelial SingleQuots^TM^ Kit (LONZA, Basel, Switzerland, Cat. No. CC–4147) was added to the BM, resulting in a proangiogenic medium including 5% Fetal Bovine Serum, 0.4% Fibroblast Growth Factor–B, 0.1% Vascular Endothelial Growth Factor, 0.1% Epidermal Growth Factor, 0.1% Insulin–like Growth Factor 1, 0.1% Ascorbic Acid, 0.1% Gentamicin sulfate–Amphotericin B and 0.04% Hydrocortisone. Refreshing of media was executed twice weekly.

### *In vitro* angiogenesis assay

12 wells of each 24-well-culture plate (Corning Life Sciences, Amsterdam, Netherlands, Cat. No. 3738) were covered with 0,5μl gelatine (Sigma Aldrich, St. Louis, MO, USA, Cat. No. G6144, 1,5% in PBS) for 20 minutes, before ECs of both batches were seeded, respectively. Cells were used in third passage in a concentration of 4.5 x 10^4^ cells per well. In total 4.86 x 10^6^ cells were cultured in 108 wells per batch. ECs were incubated for up to 50 days at 37°C in a 5% CO_2_ humidified atmosphere (INCO2/1, Memmert GmbH & Co. KG, Schwabach, Germany). On the first day of cultivation, cells of both batches were divided into three groups each. One third of cultivated ECs were infected with lentiviral particles, initiating the knockdown of VIM (sh_1_, sh_2_). By using a nontargeting hairpin, the second group represented the control group (SCR_1_, SCR_2_) and the last group consisted of native cells (N_1_, N_2_). Phase–contrast microscopy was carried out using an inverted microscope (LEICA DMi8; Leica Microsystems, Wetzlar, Germany). Angiogenesis *in vitro* was quantified according to the previously established all–in–one angiogenesis assay [14–17, 37]. Therefore, two central and two marginal visual fields per well were randomly defined per coordinates on the first day of investigation. Digital pictures of each of these visual fields were taken twice a week by using LEICA MC170 HD video camera (Leica Microsystems, Wetzlar, Germany) and the imaging and analysis software Leica Application Suite X (LAS X Version 3.4.2, Leica Microsystems, Wetzlar, Germany). Based on cell morphology in the micrographs, ECs were assigned to their respective stage of angiogenesis *in vitro* (Table 1, [16]). In an entire period of 50 days of cultivation, ECs of all groups were staged at 14 investigation days. For each visual field, the sum of allocated stages of all 14 investigation days (S^group^) was calculated. Within each group in both batches, the arithmetic mean of the sums of the visual fields were computed ($\bar{S^{\mathrm{group}}}$).

**Table 1:** Definition of stages of angiogenesis *in vitro* and description of cell morphology within the different stages [16].

Stage no.	Morphology of endothelial cells
**Stage 1**	Confluent monolayer
	Polygonal shaped cells
**Stage 2**	Endothelial sprouting, late phase
	>50% elongated shaped cells
**Stage 3**	Linear side–by–side arrangement, late phase
	>50% linearly arranged cells
**Stage 4**	Networking
	Network of linearly arranged cells
**Stage 5**	Three-dimensional organisation, early phase
	Appearance of capillary–like structures (linear structures of endothelial cells with a diameter of more than 28 μm; for these structures an internal lumen was shown by electron microscopy)
**Stage 6**	Three–dimensional organisation, late phase
	All linearly arranged cells form capillary–like structures; dissolution of cell layer on the bottom

### Quantitative analysis of VIM, TPI, MAT2A, VEGFR–1 and VEGFR–2 transcripts via RT–qPCR

Cells were harvested at days 1, 5, 25 and 50 by using Hydroxyethylpiperazine Ethane Sulfonic acid, Trypsin/EDTA and Trypsin Neutralisation Solution (LONZA, Basel, Switzerland, ReagentPack^TM^ Subculture Reagents, Cat. No. CC–5034). Instantaneously after centrifugation, cell pellets were deeply frozen in liquid nitrogen. Total RNA was isolated using Total RNA Kit, peqGold (Peqlab/VWR, Darmstadt, Germany, Cat. No. 12–6834). Remaining DNA was digested via TURBO^TM^ DNase (ThermoFisher Scientific, Bremen, Germany, Cat. No. AM2238) treatment. Isolated RNA was reverse transcribed using SuperScript IV Reverse Transcriptase (ThermoFisher Scientific, Bremen, Germany, Cat. No. 18091050) and quantitative PCR was performed with Rotor–Gene 6000 (Qiagen, Hilden, Germany) and Rotor–Gene Q 2.3.5 software. All samples were run in triplicates under use of Maxima SYBR Green qPCR Master Mix (2x) (ThermoFisher Scientific, Bremen, Germany, Cat. No K0223). Based on the standard curve method of comparative quantification, a standard curve for every gene was generated to determine the amplification efficiency and the calibrator sample. Initially, four reference genes were tested. Using the GeNorm software [38], SDHA and GAPDH were identified as the most stable reference genes and were used for further normalisation. For the normalisers and the genes of interest, the C_t_ difference between the test sample and calibrator sample was calculated and normalized to a normalisation factor of the reference genes after adjusting for minute variations in amplification efficacy. The primers were designed using NCBI/Primer–BLAST and are listed in S1 Table.

### Western blot analysis

After harvesting at day 5, 15, 25 and 50 as described above, cell pellets were resuspended in M–Per^TM^ Mammalian Protein Extraction Reagent (ThermoFisher Scientific, Bremen, Germany, Cat. No. 78501) complemented with Halt^TM^ Protease and Phosphatase Inhibitor Cocktail (ThermoFisher Scientific, Bremen, Germany, Cat. No. 78440). Protein quantity was measured via bicinchoninic acid (BCA) method using Bicinchoninic Acid Kit for Protein Determination (Sigma Aldrich, St. Louis, MO, USA, Cat. No. BCA1). On each detection day, three samples of each group were collected. 20 μg protein per sample was deployed and separated by 12% sodium dodecyl sulfate–polyacrylamide gel electrophoresis, followed by electroblotting onto nitrocellulose paper. Blots were blocked in Tris–buffered saline with 0.1% Tween–20 (TBS–T) for 1 h at room temperature and incubated over night at 4°C with primary antibodies targeting VIM (DAKO, Hamburg, Germany, M7020, 1:500), TPI (Santa Cruz, Heidelberg, Germany, H–11, 1:200), MAT2A (Santa Cruz, Heidelberg, Germany, B–10, 1:200) and Actin (Novus Biologicals, Centennial, CO, USA, AC–15, 1:5,000). Actin (ACT) served as an internal control. Subsequently, blots were washed in TBST–T buffer solution. For the detection of VIM and ACT, blots were incubated with sheep anti–mouse IgG secondary antibody (GE Healthcare, Freiburg, Germany, NA9310, 1:5,000) for 2 h at room temperature. Considering TPI and MAT2A, further incubation was unnecessary due to horseradish peroxidase being already attached to both antibodies. For visualization of proteins, SignalFire^TM^ ECL Reagent (Cell Signal technology, Frankfurt, Germany, Cat No. 6883), G:BOX Chemi XX6 gel imaging system (Syngene, Cambridge, UK) and image acquisition software GeneSys (GeneSys V1.56.0, Syngene, Cambridge, UK) was used.

### Statistics

Statistical analysis was carried out using the software SPSS Statistics (SPSS Statistics 25, IBM Corporation, Armonk, NY, USA). By executing the Shapiro–Wilk test, raw data distributions were tested. Normally distributed data is expressed as mean ± standard deviation and non–normally distributed data is expressed as median ± standard error. The comparison of two independent groups was performed with Student’s *t* test for unpaired data or Mann–Whitney U test, respectively. For more than two groups, data was analysed with one–way ANOVA and post hoc Dunn–Bonferroni test. Wilcoxon rang–sum test was used to evaluate changes of two dependant variables within a group. Statistical significance was defined as *p*<0.05.

## Results

### Gene expression of VEGFR–1 and VEGFR–2

**Fig 1:**
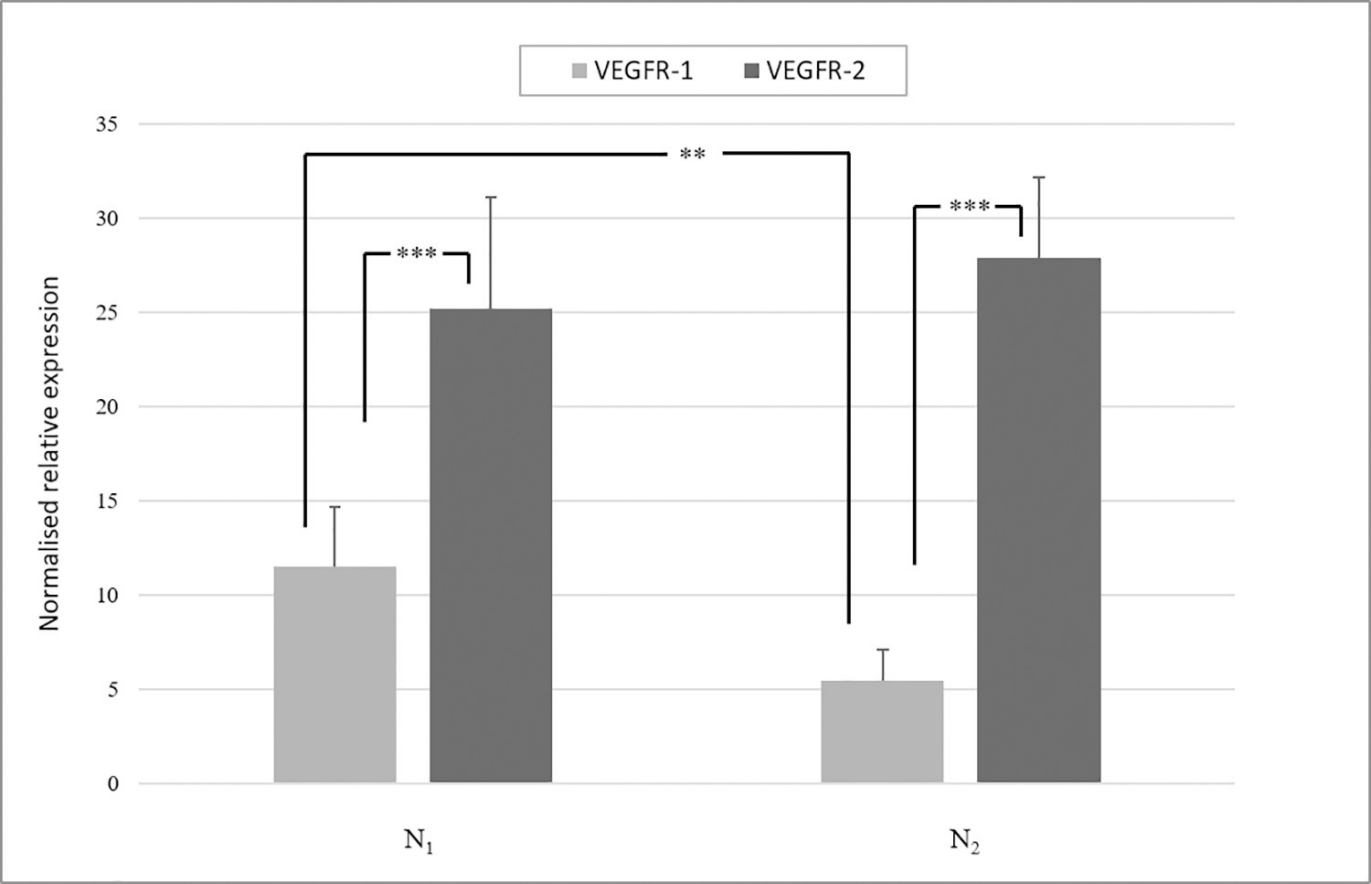
Gene expression of VEGFR–1 and VEGFR–2 in HD1 and HD2. Median and standard error of gene expression of VEGFR–1 and VEGFR–2 at all points of investigation in native ECs of HD1 and HD2 are shown. VEGFR–2 was expressed significantly higher than VEGFR–1 in N_1_ and N_2_ (*p*<0.001). Between the two batches, VEGFR–2 was expressed equally, whereas VEGFR–1 was expressed significantly higher in HD1 than in HD2 (*p*<0.01). Statistical analysis was carried out using Mann–Whitney U test for unpaired data. *p<0.05, **p<0.01, ***p<0.001.

For characterization of the cell population, the gene expression of VEGFR–1 and VEGFR–2 was measured in native cells of both batches at day 1, 5, 15, 25 and 50. Median and standard error of C_t_ values of all times are shown in the S2 Table. At all points in time, N_1_ and N_2_ showed a higher expression of VEGFR–2 than VEGFR–1 (*p*<0.05). Analysing the median of VEGFR–1 and VEGFR–2 expression for all points of investigation (Fig 1), VEGFR–2 was significantly upregulated compared to VEGFR–1 in both HD1 (*p*<0.001) and HD2 (*p*<0.001). While expression of VEGFR–2 was equal between HD1 and HD2, VEGFR–1 was significantly higher expressed in HD1 than in HD2 (*p*<0.01).

### Angiogenesis *in vitro* in N1 and N2

**Fig 2:**
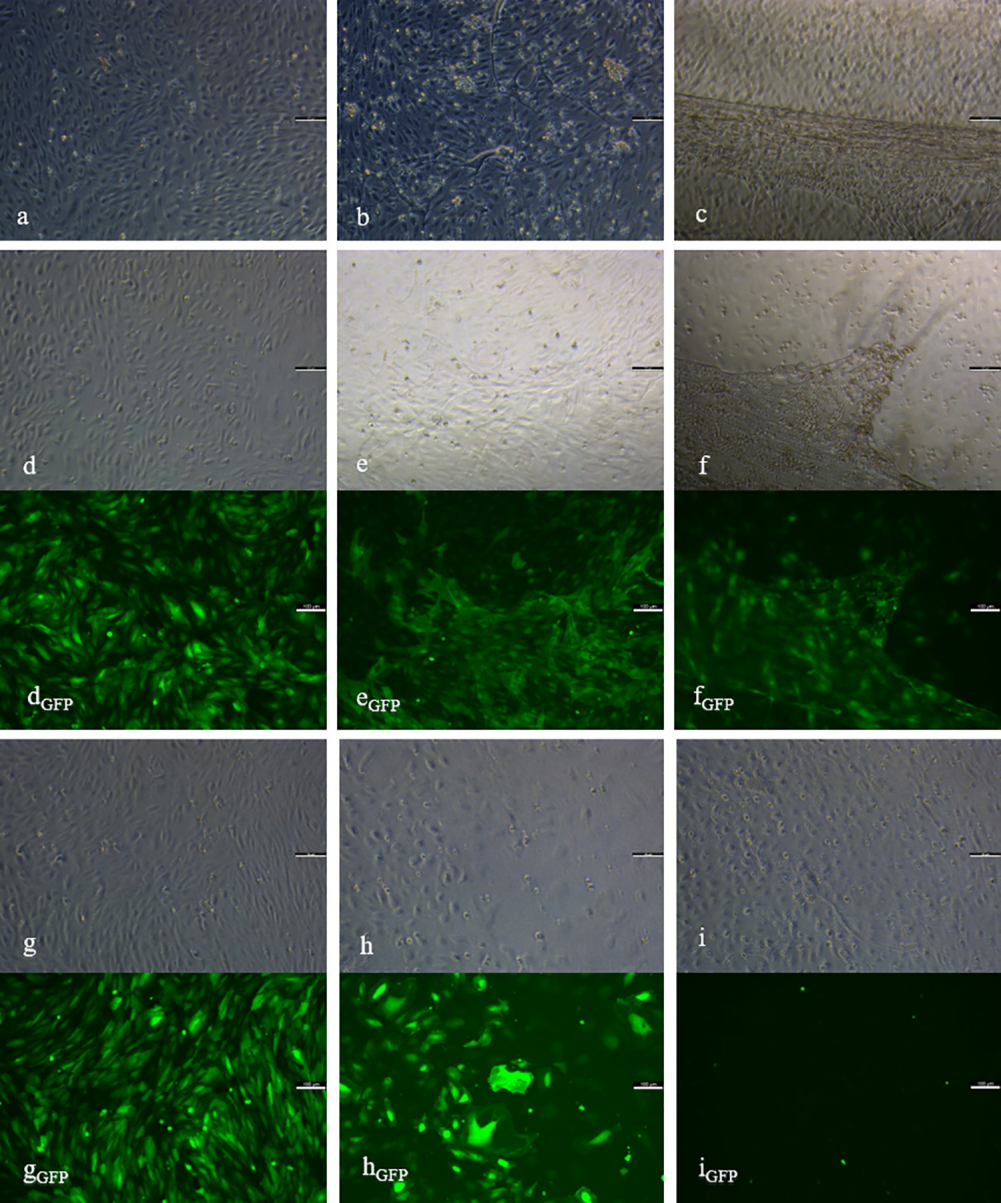
Angiogenesis *in vitro* of ECs of HD1. Native (a-c), control (d-f) and knockdown group (g-i) including the eGFP control of SCR_1_ (d_GFP_, e_GFP_, f_GFP_) and sh_1_ (g_GFP_, h_GFP_, i_GFP_) at day 5 (a, d, d_GFP_, g, g_GFP_), 25 (b, e, e_GFP_, h, h_GFP_) and 50 (c, f, f_GFP_, i, i_GFP_) of cultivation are presented. Elongated and linearly arranged cells of stage 2–3 (a, d, g) in all groups at day 5, was followed in N_1_ and SCR_1_ by networking (b, e) and three-dimensional organisation of stage 5–6 (c, f). The knockdown group showed a decrease (h), followed by an increase in cell density, ending up with networking structures of stage 4 (i). Control group showed positive eGFP control during the investigation period (d_GFP_, e_GFP_, f_GFP_), whereas sh_1_ displayed a reduction in fluorescent signals (g_GFP_, h_GFP_, i_GFP_). Scale bars = 100 μm.

**Fig 3:**
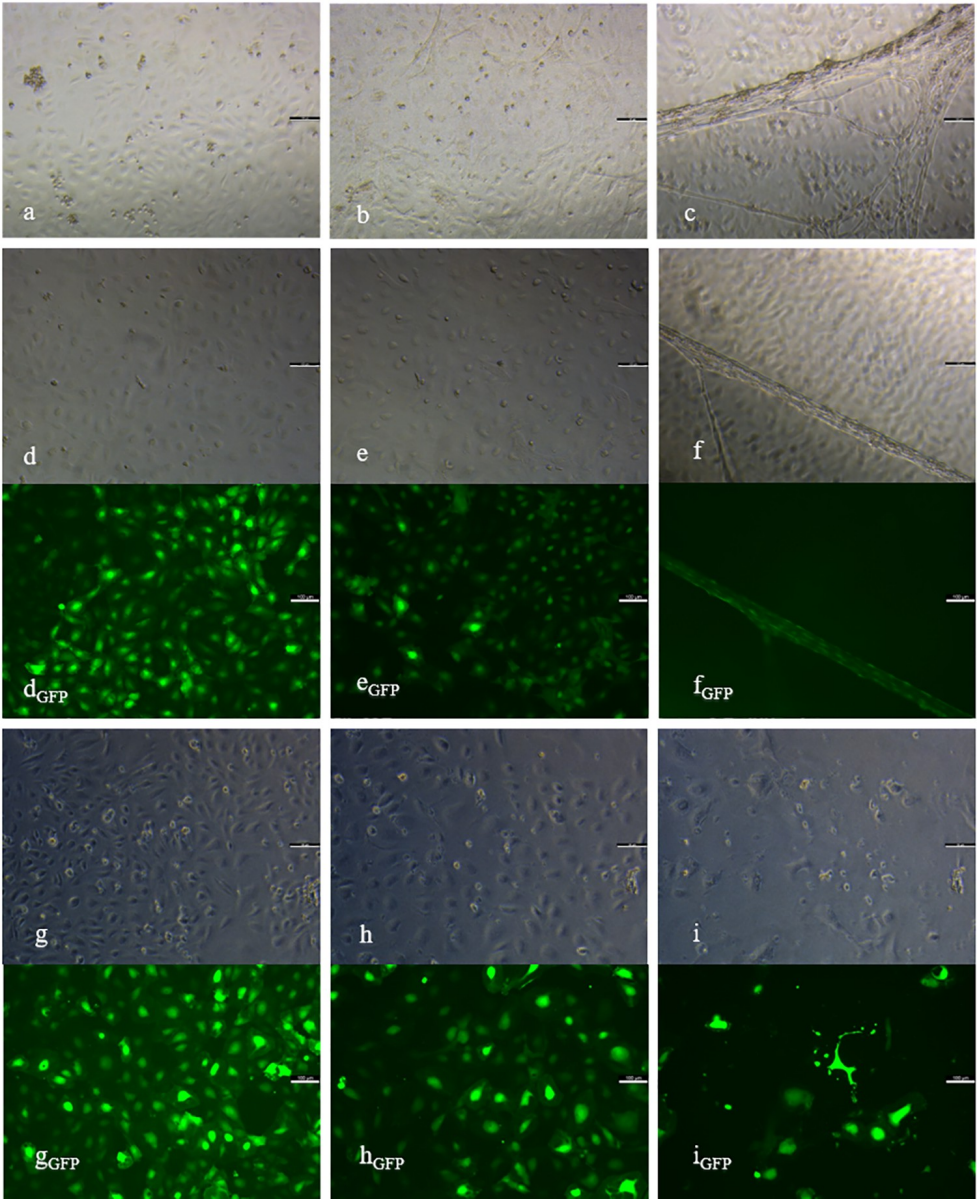
Angiogenesis *in vitro* of ECs of HD2. Native (a-c), control (d-f) and knockdown group (g-i) of HD2 including eGFP control of SCR_2_ (d_GFP_, e_GFP_, f_GFP_) and sh_2_ (g_GFP_, h_GFP_, i_GFP_) at day 5 (a, d, d_GFP_, g, g_GFP_), 25 (b, e, e_GFP_, h, h_GFP_) and 50 (c, f, f_GFP_, i, i_GFP_) of cultivation. Elongated shaped cells of stage 2 (a, d, g) in all groups at day 5, was followed in N_2_ and SCR_2_ by networking (b, e) and three-dimensional organisation in stage 5–6 (c, f). The knockdown group showed a persistent decrease in cell density (h, i). Control group showed positive eGFP control during the investigation period (d_GFP_, e_GFP_, f_GFP_), whereas sh_2_ displayed a reduction in fluorescent signals (g_GFP_, h_GFP_, i_GFP_). Scale bars = 100 μm.

In the beginning of cultivation, native cells of HD1 showed a high cell density (Fig 2A). At day 8, already more than 50% of N_1_ were linearly arranged, representing stage 3. A three–dimensional organisation, which appears in stage 5, was visible from day 25 onwards (Fig 2B). These cells were able to run through the whole angiogenic cascade chronologically in a total of 29 days, resulting in the formation of capillary–like structures and the dissolution from the bottom of the cell culture in stage 6 (Fig 2C). In contrast, native cells of HD2 displayed a lower cell density in the beginning of cultivation and less sprouting activity (Fig 3A). Therefore, more linear side–by–side arrangement was observed. By the networking of linearly arranged cells, N_2_ entered stage 4 at day 18. Finally, cells of HD2 reached stage 6 after 25 days of cultivation (Fig 3B and 3C). For quantification of angiogenesis *in vitro*, mean values and standard deviations of sums of assigned stages of angiogenesis (S) were evaluated and compared by using one–way ANOVA and Dunn–Bonferroni post hoc test. N_1_ reached a value of $\bar{S^{N_{1}}}$ = 56.9 ± 4.3 and N_2_ of $\bar{S^{N_{2}}}$ = 50.5 ± 6.5. By comparing the values between the batches, it was shown that $\bar{S^{N_{2}}}$ is significantly lower than $\bar{S^{N_{1}}}$ (*p*<0.001).

### Angiogenesis *in vitro* in SCR1 and SCR2

The high density of cells of SCR_1_ was similar to N_1_ at the beginning of cultivation (Fig 2D). SCR_1_ reached stage 3 after 11 days and stage 5 from day 29 onwards (Fig 2E). After a chronological course of all stages, SCR_1_ entered stage 6 after 32 days (Fig 2F). By detecting eGFP as an infection control, the fluorescent signal in SCR_1_ showed a constant infection until the end of cultivation (Fig 2D_GFP_–2F_GFP_). Cells of SCR_2_ showed a lower cell density in the beginning of cultivation in comparison to SCR_1_ (Fig 3D). During the course of angiogenesis *in vitro*, less sprouting but more linear side–by–side arrangement was observed. Cells of the control group entered stage 4 from day 22 onwards (Fig 3E). At day 18, 25, 32 and 39, cells of SCR_2_ were less differentiated than native cells (*p*<0.05), whereas no more difference was detectable at day 50. Finally, cells of SCR_2_ were able to undergo the whole angiogenic cascade until day 43 (Fig 3F). SCR_2_ displayed a constant fluorescent signal over the cultivation period (Fig 3D_GFP_–3F_GFP_). By comparing the sums of assigned stages of angiogenesis, it was shown that $\bar{S^{{SCR}_{2}}}$($\bar{S^{{SCR}_{2}}}$ = 49.1 ± 6.6) is significantly lower than $\bar{S^{{SCR}_{1}}}$($\bar{S^{{SCR}_{1}}}$ = 55.1 ± 5.6, *p*<0.001).

### Angiogenesis *in vitro* in sh1 and sh2

In the beginning of cultivation, cell density was high in sh_1_ (Fig 2G). However, from day 15 onwards a rapid and progressive decline in cell density was visible, with cells remaining in early stages (Fig 2H). At day 29 and following, an increase in cell density was visible. Consequently, cells entered stage 4, visible via the networking of linearly arranged cells. Cells of sh_1_ did not enter late stages of angiogenesis *in vitro* during the cultivation period of 50 days (Fig 2I). Detection of eGFP revealed a steady decline over the whole cultivation period. At day 50, eGFP was not verifiable anymore (Fig 2G_GFP_–2I_GFP_). While displaying a lower cell density than sh_1_ at day one of cultivation, the knockdown of VIM in HD2 resulted in a constant increase in cell death, beginning at day 8 (Fig 3G and 3H). Due to the persistent decrease in cell number, staging of angiogenesis was not feasible from day 36 (Fig 3I). Cells of sh_2_ showed networking but no further differentiation. In sh_2_, cell death was also detected via eGFP (Fig 3G_GFP_–3I_GFP_). The time–dependant mean values and standard deviations of assigned stages are shown in S3 Table and the course of angiogenesis *in vitro* for all groups of both batches (N_1_, SCR_1_, sh_1_, N_2_, SCR_2_, sh_2_) in S1 Fig. By comparing the values of assigned stages of angiogenesis between both knockdown groups, it was shown that cells of sh_2_ reached a sum of $\bar{S^{{sh}_{2}}}$ = 33.5 ± 10.1 and thus a significant lower value than sh_1_ resulting in $\bar{S^{{sh}_{1}}}$ = 40.6 ± 9.1 (*p*<0.001). Further analysis between groups of the same batch showed, that values of N_1_ and SCR_1_ were significantly higher than sh_1_ (*p*<0.001). In HD2, cells of the native group and the control group reached significantly higher values than sh_2_ (*p*<0.001). In both batches, native and the control groups did not show differences.

### Expression of VIM, TPI and MAT2A in N1 and N2

**Fig 4:**
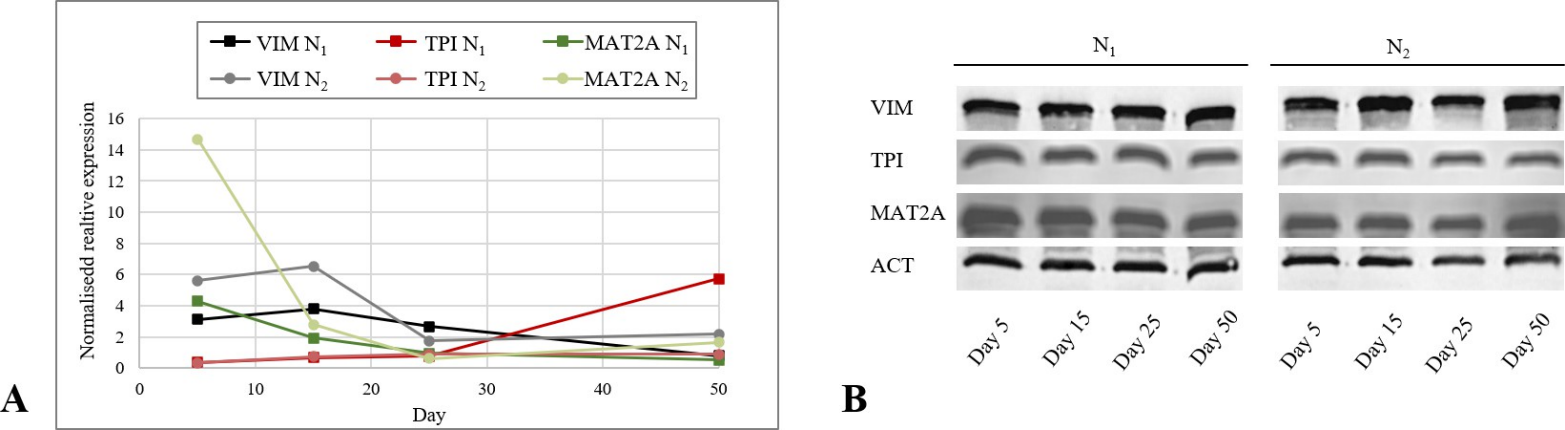
mRNA and protein expression of VIM, TPI and MAT2A in native cells. **A.** Expression of VIM, TPI and MAT2A in native endothelial cells of both batches during the angiogenic cascade. Decreasing expression in VIM and MAT2A and increasing in TPI was statistically proven under the use of Wilcoxon rang sum test (*p*<0.05). **B.** Western blot analysis of VIM, TPI and MAT2A in native cells of HD1 and HD2 at day 5, 15, 25 and 50. ACT was used as internal control. *p<0.05, **p<0.01, ***p<0.001.

In order to elucidate the expression status of VIM, TPI and MAT2A in different stages of the angiogenic cascade *in vitro*, native ECs of both batches were harvested at day 5, 15, 25 and 50 and mRNA and protein levels were measured. VIM mRNA expression was initially persistent in N_1_. From day 15 onwards, the expression was downregulated significantly on each detection day (*p*<0.05). In N_2_, VIM decreased continuously at each detection day (*p*<0.05). Comparing VIM expression between both batches, N_2_ displayed a higher expression at day 5, 15 and 50 and a lower expression at day 25 (*p*<0.05, Fig 4A). On protein level, VIM was detectable in both batches at each time point similarly (Fig 4B). Between day 5 and day 15, a significant increase in TPI mRNA expression was observed in N_1_ and N_2_ (*p*<0.05), followed by a stable expression until day 25. While, N_1_ showed an uprise in TPI expression until day 50 (*p*<0.05), N_2_ remained stable. TPI on mRNA levels was similar between N_1_ and N_2_ at day 5, 15 and 25. At day 50 TPI was higher expressed in N_1_ (*p*<0.05, Fig 4A). Western blot analysis of TPI showed stable protein levels in N_1_ and N_2_ (Fig 4B). MAT2A mRNA expression in N_1_ was decreasing from day 5 to 25 (*p*<0.01) until it remained at this level. In N_2_, MAT2A showed a decline in expression between day 5 and day 25, followed by an increase until day 50 (*p*<0.05). Expression of MAT2A in N_1_ was significantly lower at day 5, 15 and 50 and higher at day 25 than in N_2_ (Fig 4A). In both batches, protein levels were shown to be stable during the angiogenic cascade (Fig 4B). Median and standard error of protein expression is provided in S4 Table.

### Expression of VIM in sh1 and sh2

**Fig 5:**
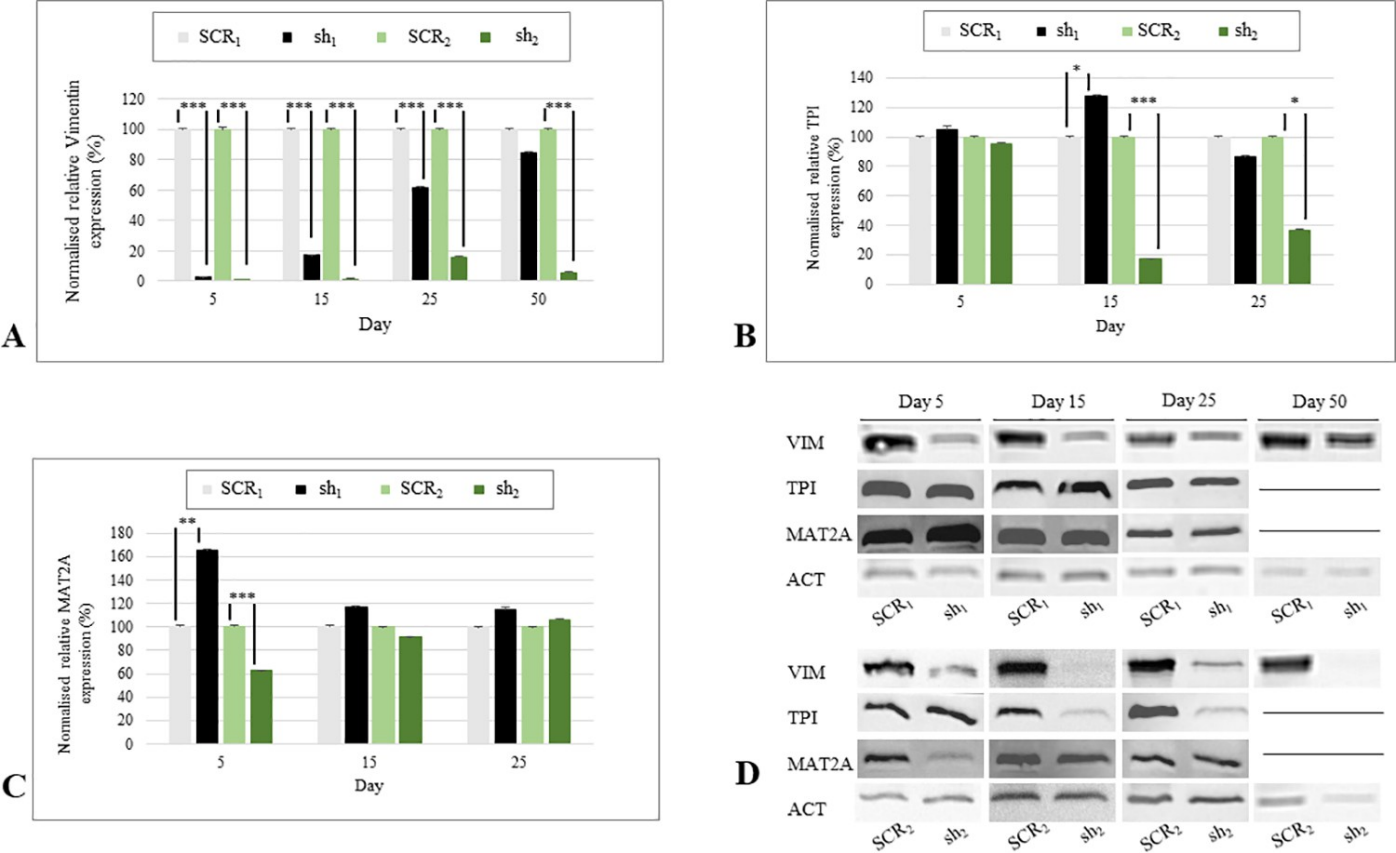
mRNA and protein expression of VIM, TPI and MAT2A in knockdown cells. **A.** Median and standard error of relative VIM mRNA expression of sh_1_, SCR_1_, sh_2_, SCR_2_. VIM was significantly downregulated in sh_1_ and sh_2_ at each investigation day (*p*<0.001), except of day 50 in sh_1._ Statistical analysis was carried out using Mann–Whitney U test for unpaired data. **B.** Relative mRNA expression of TPI of sh_1_, SCR_1_, sh_2_, SCR_2_. TPI was significantly upregulated in sh_1_ at day 15 (*p*<0.05) and downregulated in sh_2_ at day 15 (*p*<0.001), and 25 (*p*<0.05). Statistical analysis was carried out using Mann–Whitney U test for unpaired data. **C.** Median and standard error of relative MAT2A mRNA expression of sh_1_, SCR_1_, sh_2_, SCR_2_. MAT2A was significantly upregulated in sh_1_ (*p*<0.01), and downregulated in sh_2_ at day 5 (*p*<0.001)_._ Statistical analysis was carried out using Mann–Whitney U test for unpaired data. **D.** Western blot analysis of VIM, TPI and MAT2A in sh_1_, SCR_1_, sh_2_, SCR_2_ at day 5, 15, 25. ACT was used as internal control. *p<0.05, **p<0.01, ***p<0.001.

In addition to eGFP detection, RT–qPCR and Western blot analysis were used as a knockdown control. On mRNA levels, it was shown that VIM expression in sh_1_ was significantly downregulated than in SCR_1_ on day 5, 15 and 25 (*p*<0.001). At day 50, no difference was measurable (Fig 5A). In addition, on protein levels, the knockdown in HD_1_ was not visible at day 50 anymore (Fig 5D). In sh_2_ the VIM mRNA and protein levels were significantly lower at all points in time (*p*<0.001, Fig 5A and 5D). Resulting in sh_1_ and sh_2_ showing a lack in knockdown efficacy based on eGFP detection, mRNA and protein levels, data of both knockdown groups regarding day 50 are excluded from further investigations. The comparison of VIM expression in sh_1_ and sh_2_ demonstrated a significantly higher mRNA expression in sh_1_ at all time points (*p*<0.01).

### Expression of TPI and MAT2A in sh1 and sh2

To evaluate expressional changes of TPI and MAT2A occurring during the knockdown, sh_1_ and sh_2_ mRNA and protein levels were compared with SCR_1_ and SCR_2_. TPI mRNA levels were higher in sh_1_ than in SCR_1_ at day 15 (*p*<0.05) and equally at day 5 and 25, whereas sh_2_ displayed a decreased expression at day 15 (*p*<0.001) and 25 (*p*<0.05) and no difference at day 5 (Fig 5B). By comparing the knockdown groups of both batches, a higher expression of TPI was detected in sh_1_ than in sh_2_ at day 5 and 15 (*p*<0.05). Both showed equal amounts of TPI at day 25. On protein levels, TPI was detectable at each day of investigation in both batches. In HD2 protein bands matched the mRNA expression patterns of sh_2_ and SCR_2_ (Fig 5D). In contrast, the knockdown group of HD1 showed a significantly higher mRNA expression in MAT2A than SCR_1_ at day 5 (*p*<0.01), whereas sh_2_ displayed a decreased expression (*p*<0.001). At day 15 and 25, no further differences were visible (Fig 5C). These observations were confirmed on protein levels (Fig 5D). MAT2A was higher expressed in sh_2_ than in sh_1_ at day 5 (*p*<0.05) and equal at the other investigation days. Median and standard error of protein expression are provided in S4 Table.

## Discussion

Up until today, reproducibility is still an issue of concern in the research field of angiogenesis *in vitro* [6, 9–12]. ECs display an immense diversity in their behaviour during the angiogenic cascade [14–16]. It is necessary to investigate influencing factors that raise or reduce their angiogenic potency, respectively. Therefore, in this study the impact of knocking down VIM on angiogenesis *in vitro* was explored, meanwhile expressional changes in TPI and MAT2A were detected. Furthermore, native expression profiles of VIM, TPI and MAT2A were determined during the whole angiogenic cascade *in vitro*.

Native groups of HD1 and HD2 were able to undergo every stage of angiogenesis chronologically, resulting in the classification of both batches as being angiogenic ECs. By comparing the mRNA expression patterns of VEGFR–1 and –2 between both batches, it was notifiable that VEGFR–1 was significantly higher expressed in HD1 than in HD2. While VEGFR–2 is predominantly expressed in tip cells, higher levels of VEGFR–1 mRNA are detectable in stalk cells [4, 16, 39, 40]. Therefore, it can be supposed, that the cell population of HD1 comprised a higher amount of stalk cells than the cell culture of HD2. During the course of angiogenesis, stalk cells are responsible to elongate the sprout by extensive proliferation [4, 41]. Based on the higher proliferative character of HD1, N_1_ showed a greater cell density at the beginning of cultivation. Furthermore, it was shown that a low expression level of VEGFR–1 accelerates angiogenesis *in vitro* induced by VEGF–A [16], which was visible by N_2_ entering stage 6 earlier than N_1_. Additionally, the sum of assigned stages of angiogenesis in N_1_ ($\bar{S^{N_{1}}}$) and N_2_ ($\bar{S^{N_{2}}}$), in SCR_1_ ($\bar{S^{{SCR}_{1}}}$) and SCR_2_ ($\bar{S^{{SCR}_{2}}}$) and in sh_1_ ($\bar{S^{{sh}_{1}}}$) and sh_2_ ($\bar{S^{{sh}_{2}}}$) showed significantly higher values for all groups of HD1, most likely resulting from the described cell type populations.

In N_1_ and N_2_, VIM displayed a similar decreasing mRNA expression pattern as previously described in immature precursor cells [10, 21]. It was highest expressed in the beginning stages of angiogenesis in both batches while cells were morphologically staged to phases 2 and 3. This strengthens the assumption of VIM having an impact on early steps of the angiogenic cascade in HDMECs by influencing the cytoskeleton for adhesion, migration and sprouting [19, 22–24]. Mechanisms which are strongly related to endothelial tip cells. So is VIM recently described as a positive marker for epicardial tip cells [42]. By N_2_ inheriting a smaller amount of stalk cells within their population, this might be the reason for them displaying a higher mRNA expression of VIM than N_1_. Considering TPI, it was found to be upregulated in the beginning of the angiogenic cascade *in vitro*, followed by a stable mRNA expression in N_2_ and a final uprise in N_1_. TPI is involved in the glycolytic metabolism. In various stages of angiogenesis, e.g. proliferation, migration and tube formation, a high ATP supply is necessary [28, 43, 44], which might have led to the upregulation and constant expression of TPI. Expression levels of TPI were equal between both batches, except of a significantly higher mRNA level in N_1_ at day 50 which might be caused due to their higher proliferative character. During these steps, cells require energy for coordinating the formation of tube–like structures while maintaining barrier integrity [45] which was most likely intensified by N_1_ displaying a higher number of cells. Furthermore, MAT2A was significantly downregulated considering its mRNA expression in both batches during the angiogenic cascade, most strongly in the beginning. As previously described, MAT2A–activity induces the downregulation of the angiogenic potency of ECs and the initiation of maturation by synthesising SAM [33, 34]. By the cells reducing the expression of MAT2A, they might have intended to increase their angiogenic potency in order to differentiate and undergo the angiogenic cascade. The higher amount of stalk cells in N_1_ might have been the reason for the significantly lower expression of MAT2A in N_1_ than in N_2_. By showing an accelerative course of angiogenesis, N_2_ entered stages of maturation earlier than N_1_, which might have led to a lower MAT2A mRNA expression level at day 25 followed by a significant uprise at day 50. For validating the impact of TPI and MAT2A on the angiogenic cascade *in vitro*, further investigations must be initiated.

Over the whole cultivation period of 50 days, the infection appeared to be successful and persistent. Both, SCR_1_ and SCR_2_, were able to enter all stages of the angiogenic cascade, including eGFP positive structures. Additionally, protein levels and quantifying angiogenesis displayed no differences between N_1_ ($\bar{S^{N_{1}}}$) and SCR_1_ ($\bar{S^{{SCR}_{1}}}$), N_2_ ($\bar{S^{N_{2}}}$) and SCR_2_ ($\bar{S^{{SCR}_{2}}}$). However, a delay in entering the stages of angiogenesis *in vitro* was detectable in SCR_2_, while morphological staging of SCR_1_ was mostly equal to N_1_, concluding that the infection with viral particles might have affected SCR_2_ more intensely than SCR_1_. As previously described, lentiviral transduction can have an adverse effect on cell proliferation, depending on the target cell [46]. With SCR_2_ having less stalk cells and showing a less proliferative character, this might be the reason for them being influenced more intensely by manipulations.

The downregulation of VIM expression led to cell death of infected cells in both cultures. Via the protein Rudhira/Breast Carcinoma Amplified Seqence 3, VIM is linked to microtubules, resulting in the stabilization of the cytoskeleton, which is essential for focal adhesion and cells migration [20, 47]. ECs are adherent cells, for which it is necessary to create cell–cell and cell–matrix connections for stability, communication and differentiation [19, 48]. By knocking down VIM, ECs were most likely not able to build a stable cell layer for interaction and further developments, resulting in cell death. While cells of sh_2_ were dying progressively, sh_1_ recovered from cell loss. The higher amount of stalk cells in sh_1_ might have facilitated an increase in cell density and enabled further differentiations. It can be supposed that due to their proliferative character, sh_1_ showed a greater cell number of uninfected cells which led to a generally higher mRNA expression of VIM in sh_1_ than in sh_2_. Regarding the angiogenic cascade, a part of the infected cells of both knockdown groups were able to enter stage 4 as a maximum, suggesting that cells still owned native VIM. In sh_1_, the number of uninfected cells increased, so that no reliable interpretations about VIM were possible anymore. However, quantitation of angiogenesis stated the deceleration of angiogenesis in both batches, by the sum of assigned stages for the knockdown groups ($\bar{S^{{sh}_{1}}},\bar{S^{{sh}_{2}}}$) being significantly smaller than the control ($\bar{S^{{SCR}_{1}}},\bar{S^{{SCR}_{2}}}$) and the native group ($\bar{S^{N_{1}}},\bar{S^{N_{2}}}$). This leads to the assumption that VIM raises the angiogenic potency of HDMECs *in vitro* and is essential for cell survival.

Comparing sh_1_ and sh_2_ with SCR_1_ and SCR_2,_ sh_1_ displayed a significantly higher TPI mRNA expression than SCR_1_ at day 15. An increase in TPI was already related to a cellular stress response, in order to upregulate cellular energy metabolisms [30]. While TPI is upregulated in sh_1_, sh_2_ displayed a significant decrease in protein and mRNA levels at day 15 and 25. From day 8 onwards, a persistent death of ECs in sh_2_ was detectable. As previously described, cell death leads towards a dysregulation of metabolisms, resulting in no further metabolic activity [49]. This impact on cell metabolism might have caused the lower expression of TPI in sh_2_ in comparison with sh_1_ at day 5 and 15. Suggesting that MAT2A influences ECs via SAM-mediated methylations, the upregulation in MAT2A expression in sh_1_ at day 5 might be a support mechanism to reduce angiogenic activity. Contrarily, the cell population of sh_2_might have intended to decrease cellular SAM levels by downregulating MAT2A at day 5. It was shown that SAM inhibits mitogenic effects of growth factors, with the aim of recovering damaged cells [50].

## Conclusion

This study stated that different endothelial cell batches, which are isolated from the same tissue, acquired from the same distributor and being cultivated under the same conditions, show different morphologies during the angiogenic cascade *in vitro*. Despite being characterized as angiogenic cultures, both batches vary tremendously in their behaviour of compensating with manipulations. By knocking down of VIM, the cell population with the higher number of stalk cells was able to increase in cell density and upregulated TPI and MAT2A expression. In contrast, cell population with less stalk cells died continuously, going along with the downregulation of TPI and MAT2A expression. Generally, VIM knockdown decelerated angiogenesis *in vitro* and resulted in cell death in both batches, concluding that VIM might be an essential protein for the angiogenic cascade and cell survival of ECs. Additionally, it was shown that VIM and MAT2A were highest expressed in beginning stages of angiogenesis *in vitro*, followed by a low-level expression. Concurrently, TPI was first upregulated and subsequently stably expressed during the course of angiogenesis. In order to validate the connection between the three proteins and to determine the impact of TPI and MAT2A on angiogenesis, it is necessary to do further research, i.e. knocking down of TPI and of MAT2A. Moreover, the specific mechanisms, on how these proteins affect ECs during angiogenesis *in vitro* must be analysed.

## Supporting information

S1 Fig Stages of angiogenesis *in vitro* of HDMECs. Native groups (N_1_, N_2_), control groups (SCR_1_, SCR_2_) and knockdown groups (sh_1_, sh_2_) are presented. Mean values are calculated for 4 visual fields of 4 wells per culture at 14 detection days during a cultivation period of 50 days. Native and control groups of both batches ran through all six stages of angiogenesis chronologically. Cells of sh_1_ and sh_2_ entered stage 4 as a maximum. Sh_2_ displayed a persistence in cell death until no further staging was possible from day 36 onwards.
(TIF)

S1 Table List of primers used for overexpression (VIM^+^) and RT–qPCR. Genes of interest: qVIM, qMAT2A, qTPI, qVEGFR–1 and qVEGFR–2; reference genes: succinate dehydrogenase complex, subunit A (qSDHA), hydroxymethylebilane synthase (qHMBS), glyceraldehyde–3–phosphate dehydrogenase (qGAPDH) and TATA box binding protein (qTBP).
(DOCX)

S2 Table VEGFR–1 and VEGFR–2 expression in native ECs of HD1 and HD2. Median and standard error was evaluated at day 1, 5, 15, 25 and 50 of cultivation. By using Mann–Whitney U test for unpaired data, it was shown that VEGFR–2 was higher expressed than VEGFR–1 at every point of investigation in both cultures (p<0.05).
(DOCX)

S3 Table Morphologically assigned stages of angiogenesis *in vitro*. Mean values and standard deviation for all groups of both batches at 14 detection days over the cultivation period of 50 days are presented.
(DOCX)

S4 Table VIM, TPI and MAT2A expression. Median and standard error of VIM TPI and MAT2A expression in N_1_, SCR_1_, sh_1,_ N_2_, SCR_2_ and sh_2_ are shown for day 5, 15, 25 and 50.
(DOCX)

S1 Raw images (PDF)
