# Supplementary figures and images for: Expression of vimentin, TPI and MAT2A in human dermal microvascular endothelial cells during angiogenesis in vitro
Source: PLoS One. 2022 Apr 28;17(4):e0266774. doi: 10.1371/journal.pone.0266774 (PMC9049311; doi:10.1371/journal.pone.0266774)

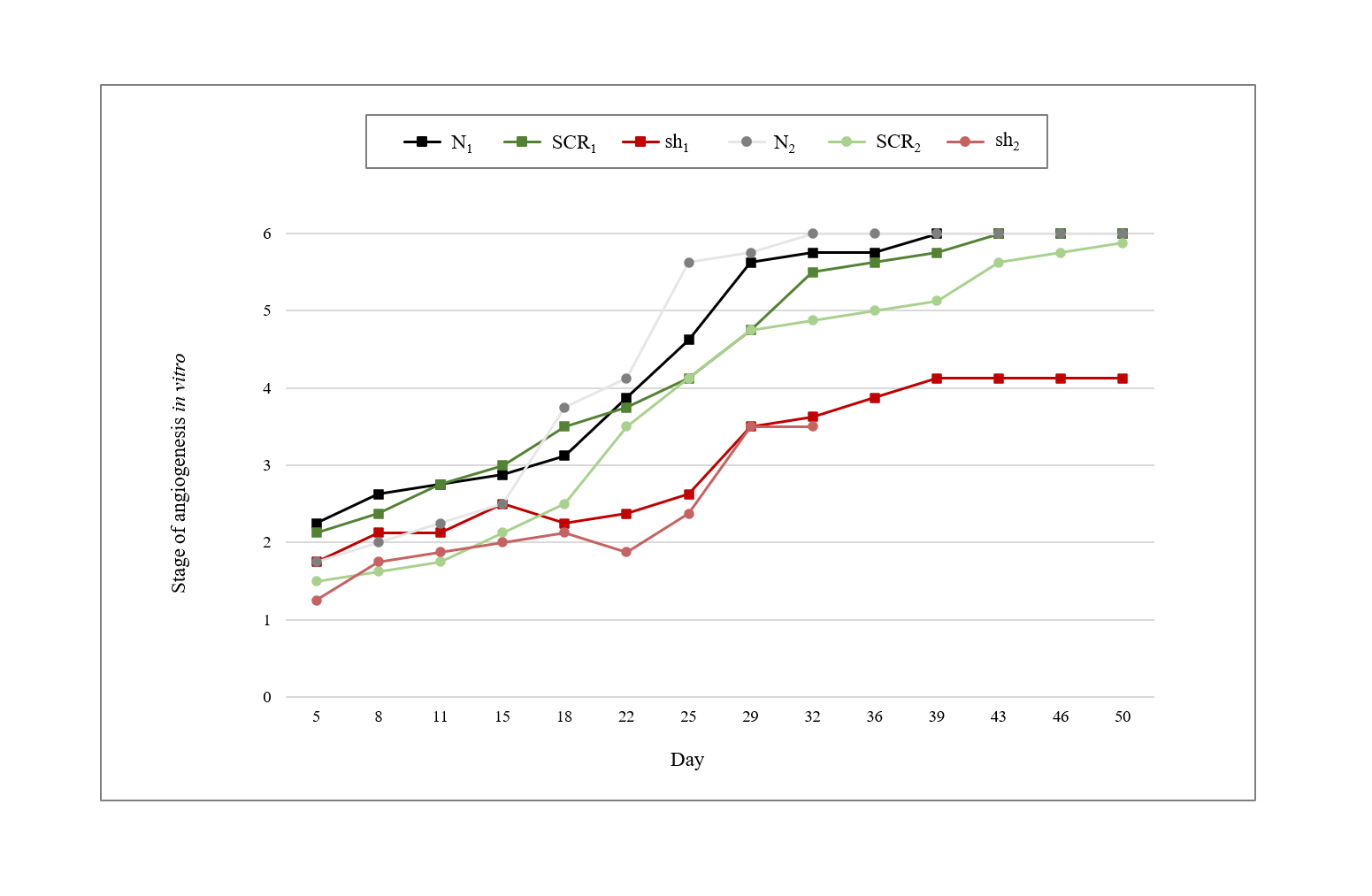

Supplement: S1 Fig — Native groups (N1, N2), control groups (SCR1, SCR2) and knockdown groups (sh1, sh2) are presented. Mean values are calculated for 4 visual fields of 4 wells per culture at 14 detection days during a cultivation period of 50 days. Native and control groups of both batches ran through all six stages of angiogenesis chronologically. Cells of sh1 and sh2 entered stage 4 as a maximum. Sh2 displayed a persistence in cell death until no further staging was possible from day 36 onwards. (TIF) [file pone.0266774.s001.tif]
