# Supplementary material for: Expression of vimentin, TPI and MAT2A in human dermal microvascular endothelial cells during angiogenesis in vitro
Source: PLoS One. 2022 Apr 28;17(4):e0266774. doi: 10.1371/journal.pone.0266774 (PMC9049311; doi:10.1371/journal.pone.0266774)
Supplement: S1 Table — Genes of interest: qVIM, qMAT2A, qTPI, qVEGFR–1 and qVEGFR–2; reference genes: succinate dehydrogenase complex, subunit A (qSDHA), hydroxymethylebilane synthase (qHMBS), glyceraldehyde–3–phosphate dehydrogenase (qGAPDH) and TATA box binding protein (qTBP). (DOCX) [file pone.0266774.s002.docx]

| **Target Gene** | **Orientation** | **Primer sequence (5’ – 3’)** | **Amplicon length (bp)** | **Annealing Temperature (°C)** |
| --- | --- | --- | --- | --- |
| **VIM^+^** | forward | GCGGGATCCGCCACCATGTCCACCAGGTCCGTGTCC | 101 | 69 |
|  | reverse | GCGGAATTCAATTCAAGGTCATCGTGATG |  |  |
| **qVIM** | forward | GGCACGTCTTGACCTTGAAC | 110 | 64 |
|  | reverse | GTTCCTGAATCTGAGCCTGC |  |  |
| **qTPI** | forward | TGGCATCACTGAGAAGGTTG | 122 | 63 |
|  | reverse | TTGCAGTCTTGCCAGTACCA |  |  |
| **qMAT2A** | forward | CTGGCAGAACTACGCCGTAATG | 116 | 66 |
|  | reverse | GTGTGGACTCTGATGGGAAGCA |  |  |
| **qSDHA** | forward | CAAACTCGCTCTTGGACCTG | 118 | 64 |
|  | reverse | ACAGATTCTTCCCCAGCGTT |  |  |
| **qHMBS** | forward | TGCCAGAGAAGAGTGTGGTG | 101 | 62 |
|  | reverse | GAGGTTTCCCCGAATACTCC |  |  |
| **qGAPDH** | forward | ACACCCACTCCTCCACCTTT | 99 | 62 |
|  | reverse | TGCTGTAGCCAAATTCGTTG |  |  |
| **qTBP** | forward | GAGCTGTGATGTGAAGTTTCC | 118 | 61 |
|  | reverse | TCTGGGTTTGATCATTCTGTAG |  |  |
| **qVEGFR–1 [16]** | forward | GACCTGGAGTTACCCTGATGAAA | 76 | 60 |
|  | reverse | GGCATGGGAATTGCTTTGG |  |  |
| **qVEGRF–2 [16]** | forward | CACCACTCAAACGCTGACATGTA | 95 | 60 |
|  | reverse | GCTCGTTGGCGCACTCTT |  |  |
