# Supplementary material for: Expression of vimentin, TPI and MAT2A in human dermal microvascular endothelial cells during angiogenesis in vitro
Source: PLoS One. 2022 Apr 28;17(4):e0266774. doi: 10.1371/journal.pone.0266774 (PMC9049311; doi:10.1371/journal.pone.0266774)
Supplement: S2 Table — Median and standard error was evaluated at day 1, 5, 15, 25 and 50 of cultivation. By using Mann–Whitney U test for unpaired data, it was shown that VEGFR–2 was higher expressed than VEGFR–1 at every point of investigation in both cultures (p<0.05). (DOCX) [file pone.0266774.s003.docx]

|  | **Day 1**  **VEGFR–1 VEGFR–2** | | **Day 5**  **VEGFR–1 VEGFR–2** | | **Day 15**  **VEGFR–1 VEGFR–2** | | **Day 25**  **VEGFR–1 VEGFR–2** | | **Day 50**  **VEGFR–1 VEGFR–2** | |
| --- | --- | --- | --- | --- | --- | --- | --- | --- | --- | --- |
| **N_1_** | 25.92  ± 3.14 | 54.42  ± 4.43 | 12.37  ± 5.70 | 20.98  ± 8.82 | 11.63  ± 1.16 | 24.47  ± 2.05 | 6.16  ± 2.21 | 14.72  ± 4.55 | 7.44  ± 1.61 | 102.94  ± 5.05 |
| **N_2_** | 1.55  ± 0.70 | 8.56  ± 1.77 | 8.26  ± 1.90 | 40.26  ± 5.24 | 10.29  ± 0.83 | 62.06  ± 3.69 | 5.24  ± 1.34 | 18.33  ± 3.67 | 5.22  ± 1.11 | 16.09  ± 2.30 |
