# Supplementary material for: Expression of vimentin, TPI and MAT2A in human dermal microvascular endothelial cells during angiogenesis in vitro
Source: PLoS One. 2022 Apr 28;17(4):e0266774. doi: 10.1371/journal.pone.0266774 (PMC9049311; doi:10.1371/journal.pone.0266774)
Supplement: S3 Table — Mean values and standard deviation for all groups of both batches at 14 detection days over the cultivation period of 50 days are presented. (DOCX) [file pone.0266774.s004.docx]

|  | **Day 4** | **Day 8** | **Day 11** | **Day 15** | **Day 18** | **Day 22** | **Day 25** | **Day 29** | **Day 32** | **Day 36** | **Day 39** | **Day 43** | **Day 46** | **Tag50** |
| --- | --- | --- | --- | --- | --- | --- | --- | --- | --- | --- | --- | --- | --- | --- |
| **N_1_** | 2.25 | 2.63 | 2.75 | 2.88 | 3.13 | 3.88 | 4.63 | 5.63 | 5.75 | 5.75 | 6.00 | 6.00 | 6.00 | 6.00 |
|  | ± 0.46 | ± 0.52 | ± 0.46 | ± 0.35 | ± 0.64 | ± 0.35 | ± 0.52 | ± 0.52 | ± 0.46 | ± 0.46 | ± 0.00 | ± 0.00 | ± 0.00 | ± 0.00 |
| **SCR_1_** | 2.13 | 2.38 | 2.75 | 3.00 | 3.50 | 3.75 | 4.13 | 4.75 | 5.50 | 5.63 | 5.75 | 6.00 | 6.00 | 6.00 |
|  | ± 0.35 | ± 0.52 | ± 0.46 | ± 0.53 | ± 0.76 | ± 0.46 | ± 0.35 | ± 0.46 | ± 0.53 | ± 0.52 | ± 0.46 | ± 0.00 | ± 0.00 | ± 0.00 |
| **sh_1_** | 1.75 | 2.13 | 2.13 | 2.50 | 2.25 | 2.38 | 2.63 | 3.50 | 3.63 | 3.88 | 4.13 | 4.13 | 4.13 | 4.13 |
|  | ± 0.46 | ± 0.35 | ± 0.35 | ± 0.53 | ± 0.46 | ± 0.52 | ± 0.74 | ± 0.76 | ± 0.74 | ± 0.83 | ± 0.35 | ± 0.35 | ± 0.35 | ± 0.35 |
| **N_2_** | 1.75 | 2.00 | 2.25 | 2.50 | 3.75 | 4.13 | 5.63 | 5.75 | 6.00 | 6.00 | 6.00 | 6.00 | 6.00 | 6.00 |
|  | ± 0.46 | ± 0.00 | ± 0.71 | ± 0.93 | ± 0.71 | ± 0.64 | ± 0.74 | ± 0.46 | ± 0.00 | ± 0.00 | ± 0.00 | ± 0.00 | ± 0.00 | ± 0.00 |
| **SCR_2_** | 1.50 | 1.63 | 1.75 | 2.13 | 2.50 | 3.50 | 4.13 | 4.75 | 4.88 | 5.00 | 5.13 | 5.63 | 5.75 | 5.88 |
|  | ± 0.53 | ± 0.52 | ± 0.46 | ± 0.83 | ± 0.93 | ± 0.93 | ± 0.99 | ± 0.46 | ± 0.35 | ± 0.00 | ± 0.35 | ± 0.52 | ± 0.46 | ± 0.35 |
| **sh_2_** | 1.25 | 1.75 | 1.88 | 2.00 | 2.13 | 1.88 | 2.38 | 3.50 | 3.50 | – | – | – | – | – |
|  | ± 0.46 | ± 0.46 | ± 0.64 | ± 0.53 | ± 0.64 | ± 0.64 | ± 0.52 | ± 0.93 | ± 0.93 | – | – | – | – | – |
