# Supplementary material for: Expression of vimentin, TPI and MAT2A in human dermal microvascular endothelial cells during angiogenesis in vitro
Source: PLoS One. 2022 Apr 28;17(4):e0266774. doi: 10.1371/journal.pone.0266774 (PMC9049311; doi:10.1371/journal.pone.0266774)
Supplement: S4 Table — Median and standard error of VIM TPI and MAT2A expression in N1, SCR1, sh1, N2, SCR2 and sh2 are shown for day 5, 15, 25 and 50. (DOCX) [file pone.0266774.s005.docx]

|  | **Day 5** | **Day 15** | **Day 25** | **Day 50** |
| --- | --- | --- | --- | --- |
| **VIM** | 3.11 ± 1.07 | 3.80 ± 0.67 | 2.67 ± 0.64 | 0.79 ± 0.99 |
| **N_1_  TPI** | 0.37 ± 0.46 | 0.68 ± 0.42 | 0.76 ± 0.67 | 12.15 ± 3.87 |
| **MAT2A** | 4.30 ± 1.13 | 1.93 ± 0.73 | 0.94 ± 1.79 | 0.51 ± 1.89 |
|  |  |  |  |  |
| **VIM** | 3.77 ± 1.25 | 1.72 ± 1.08 | 1.77 ± 0.55 | 0.98 ± 0.56 |
| **SCR_1_  TPI** | 0.31 ± 0.54 | 0.73 ± 0.42 | 0.97 ± 0.87 | 7.99 ± 1.62 |
| **MAT2A** | 6.94 ± 1.62 | 0.48 ± 0.96 | 0.54 ± 0.35 | 2.09 ± 0.97 |
|  |  |  |  |  |
| **VIM** | 0.12 ± 0.26 | 0.30 ± 0.41 | 1.04 ± 0.44 | 0.69 ± 0.46 |
| **sh_1_  TPI** | 0.85 ± 1.05 | 0.93 ± 0.57 | 0.66 ± 0.54 | X |
| **MAT2A** | 10.56 ± 1.76 | 0.56 ± 0.97 | 0.63 ± 1.04 | X |
|  |  |  |  |  |
| **VIM** | 5.60 ± 1.25 | 6.53 ± 1.12 | 1.73 ± 0.49 | 2.18 ± 0.59 |
| **N_2_  TPI** | 0.34 ± 0.21 | 0.74 ± 0.46 | 0.89 ± 0.31 | 0.87 ± 0.41 |
| **MAT2A** | 14.67 ± 1.28 | 2.79 ± 0.67 | 0.63 ± 0.46 | 1.65 ± 1.81 |
|  |  |  |  |  |
| **VIM** | 7.01 ± 1.35 | 3.59 ± 0.87 | 2.12 ± 0.70 | 1.87 ± 0.60 |
| **SCR_2_ TPI** | 0.22 ± 0.29 | 0.92 ± 0.51 | 1.08 ± 0.90 | 0.95 ± 0.42 |
| **MAT2A** | 12.93 ± 1.32 | 1.57 ± 0.78 | 0.41 ± 0.37 | 0.95 ± 0.31 |
|  |  |  |  |  |
| **VIM** | 0.04 ± 0.10 | 0.06 ± 0.24 | 0.34 ± 0.30 | 0.11 ± 0.22 |
| **sh_2_  TPI** | 0.21 ± 0.28 | 0.16 ± 0.32 | 0.35 ± 0.74 | X |
| **MAT2A** | 8.13 ± 1.46 | 1.44 ± 0.71 | 0.44 ± 0.46 | X |
