# Supplementary material for: Expression of vimentin, TPI and MAT2A in human dermal microvascular endothelial cells during angiogenesis in vitro
Source: PLoS One. 2022 Apr 28;17(4):e0266774. doi: 10.1371/journal.pone.0266774 (PMC9049311; doi:10.1371/journal.pone.0266774)
Supplement: S1 Raw images — (PDF) [file pone.0266774.s006.pdf]

Detection of Vimentin and Actin for all native groups at day 5, 15, 25 and 50

- Aim: Comparison of protein expression of VIM, TPI and MAT2A in N_1 and N_2 respectively over the time
- Same samples were inserted identically in both gels (20µg) and detected in the same run

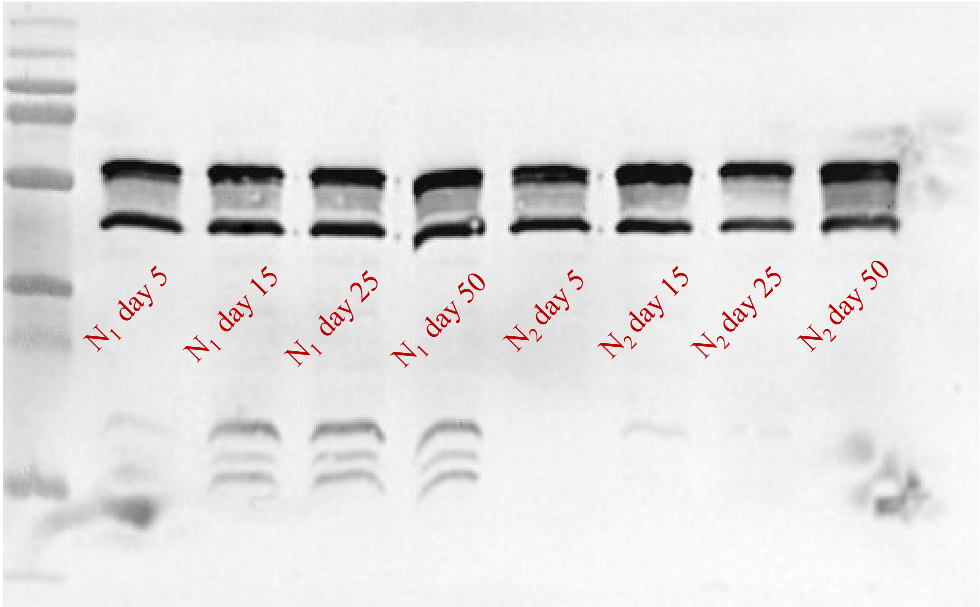

Vimentin (~57kDa)

Actin (~42kDa)

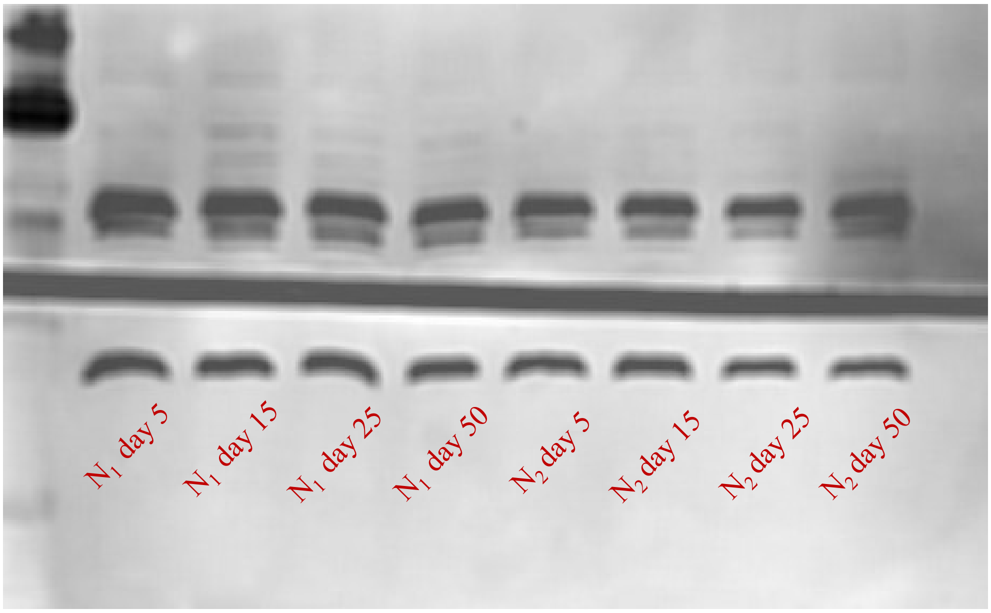

MAT2A (~43kDa)

Cut line (~35kDa)

TPI (~30kDa)

Detection of Vimentin and Actin for N, SCR and sh of HD1 in triplicates

- Aim: Comparison of protein expression of VIM in sh_1 and SCR_1 at each detection day separately
- 20µg of each sample was used

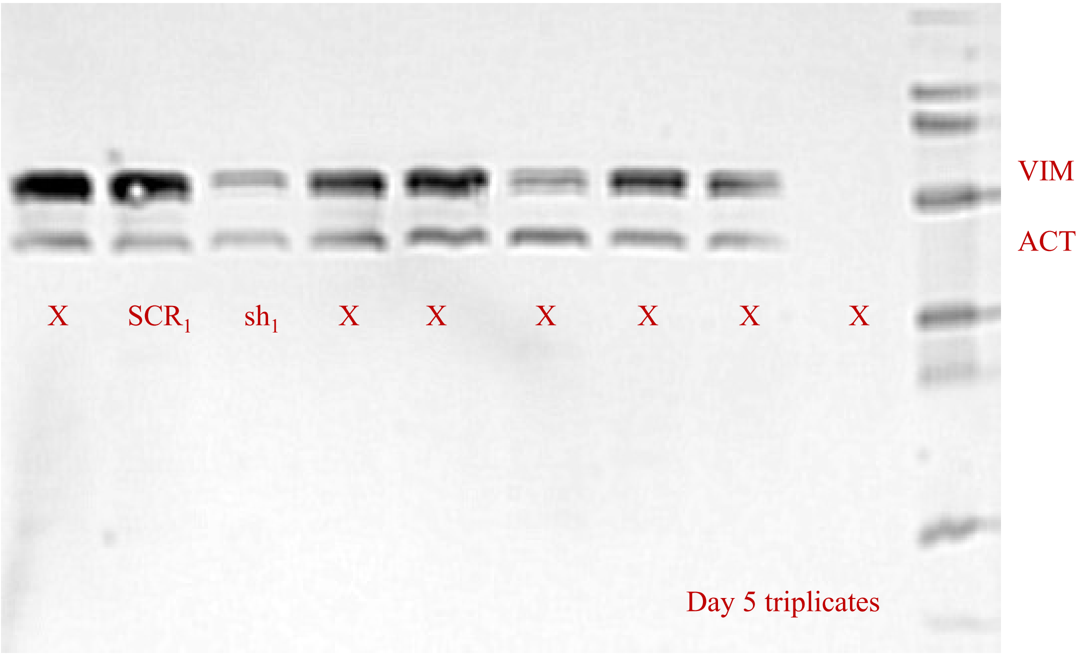

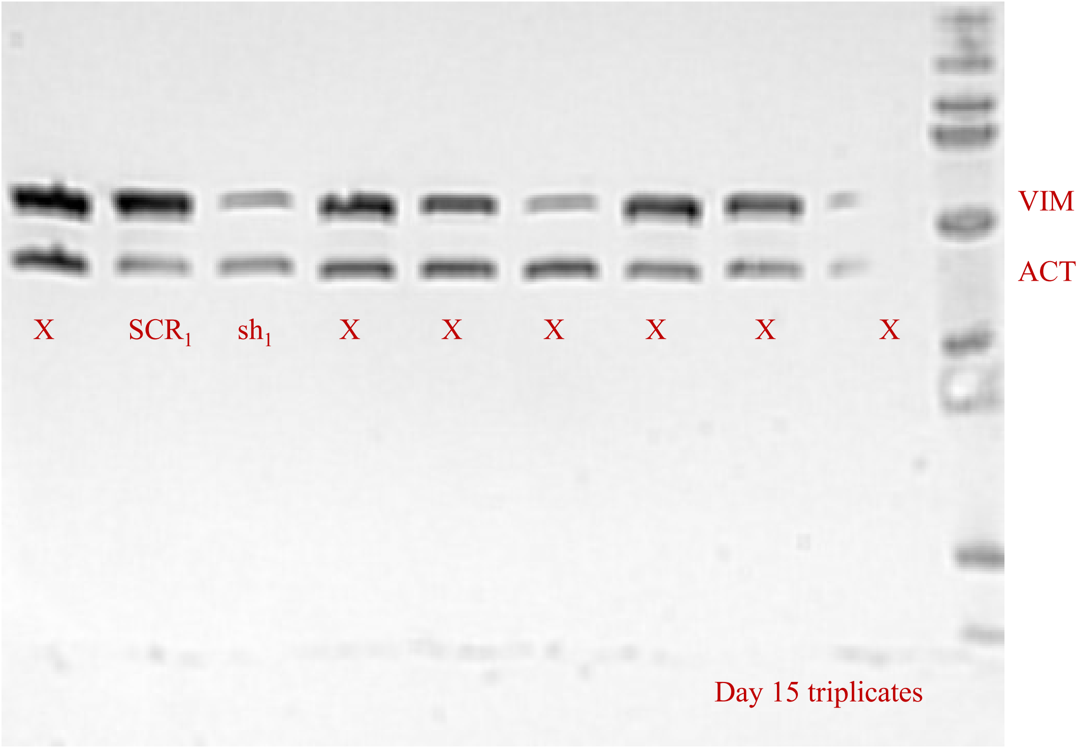

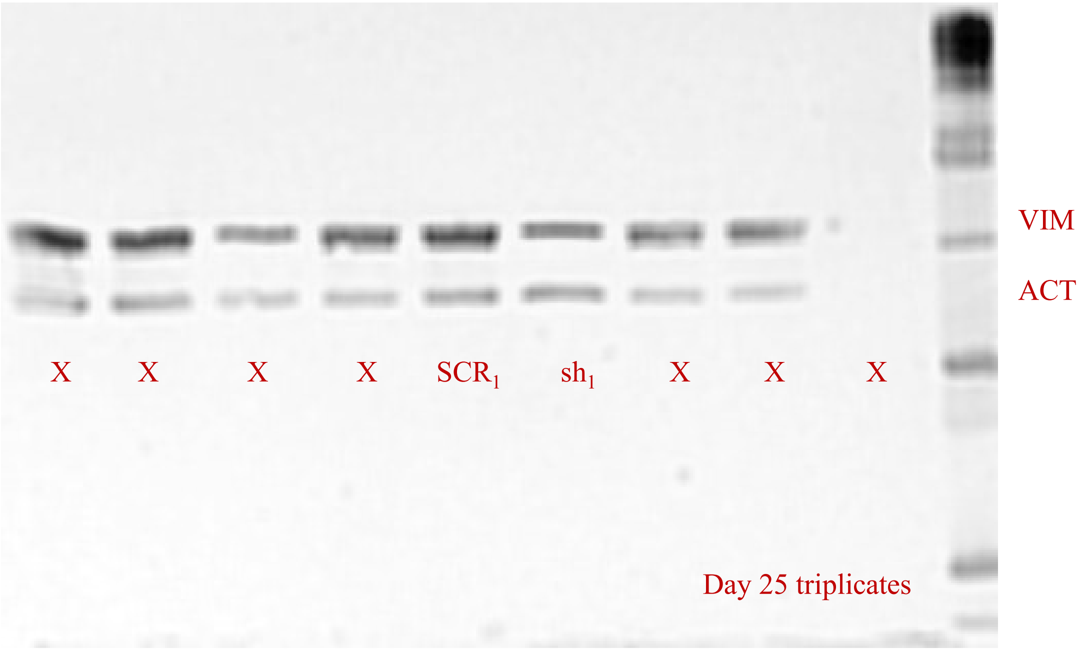
VIM
ACT
X
X
X
X
SCR$_1$
sh$_1$
X
X
X
Day 25 triplicates

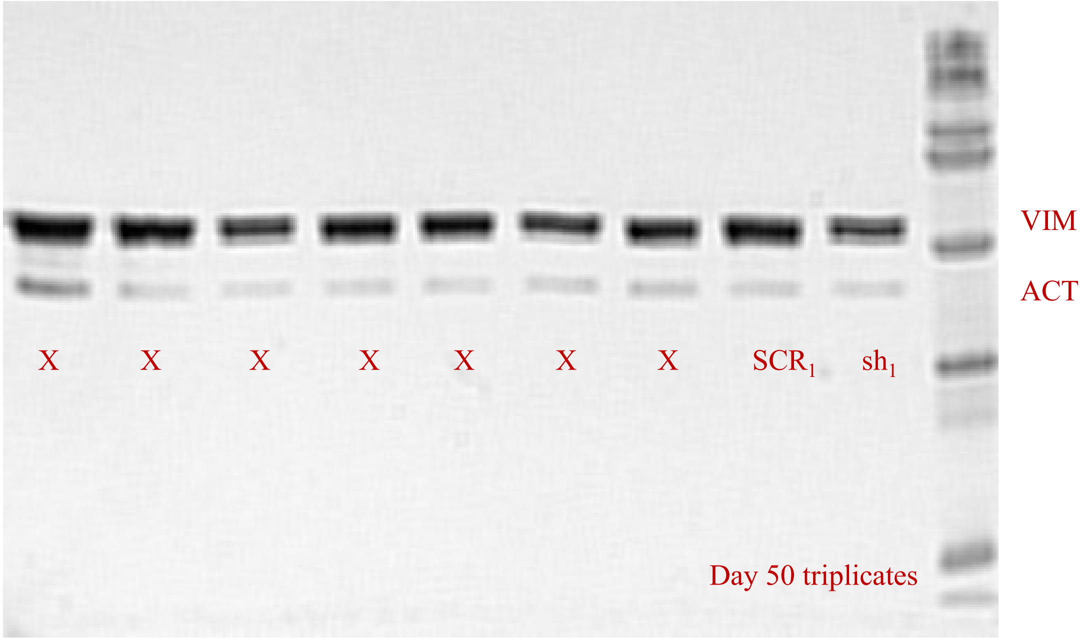
VIM
ACT
X
X
X
X
X
X
X
SCR$_1$
sh$_1$
Day 50 triplicates

Detection of Vimentin and Actin for N, SCR and sh of HD2 in triplicates

- Aim: Comparison of protein expression of VIM in sh_2 and SCR_2 at each detection day separately
- 20µg of each sample was used

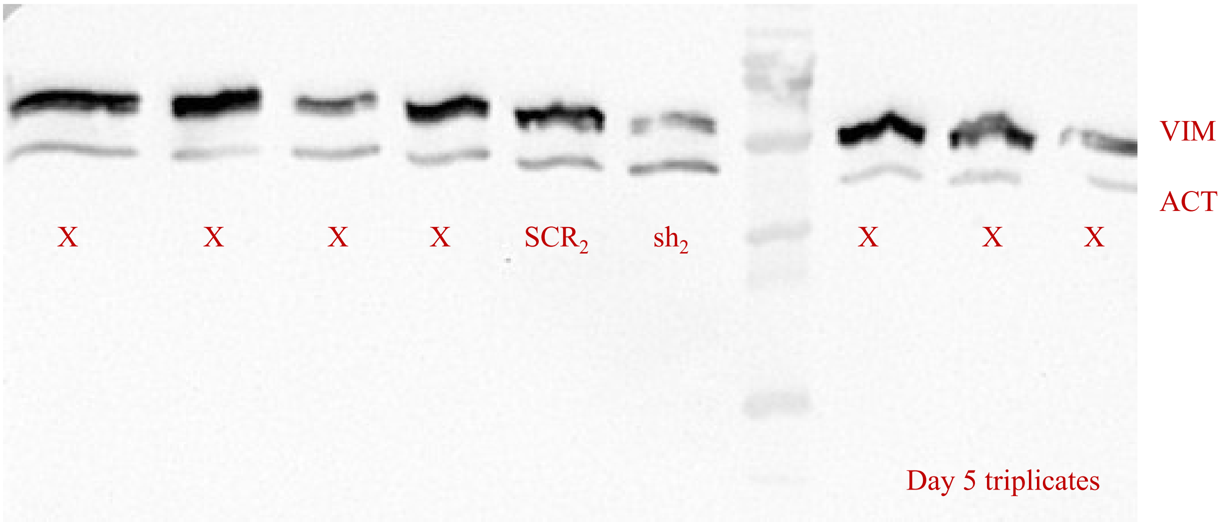

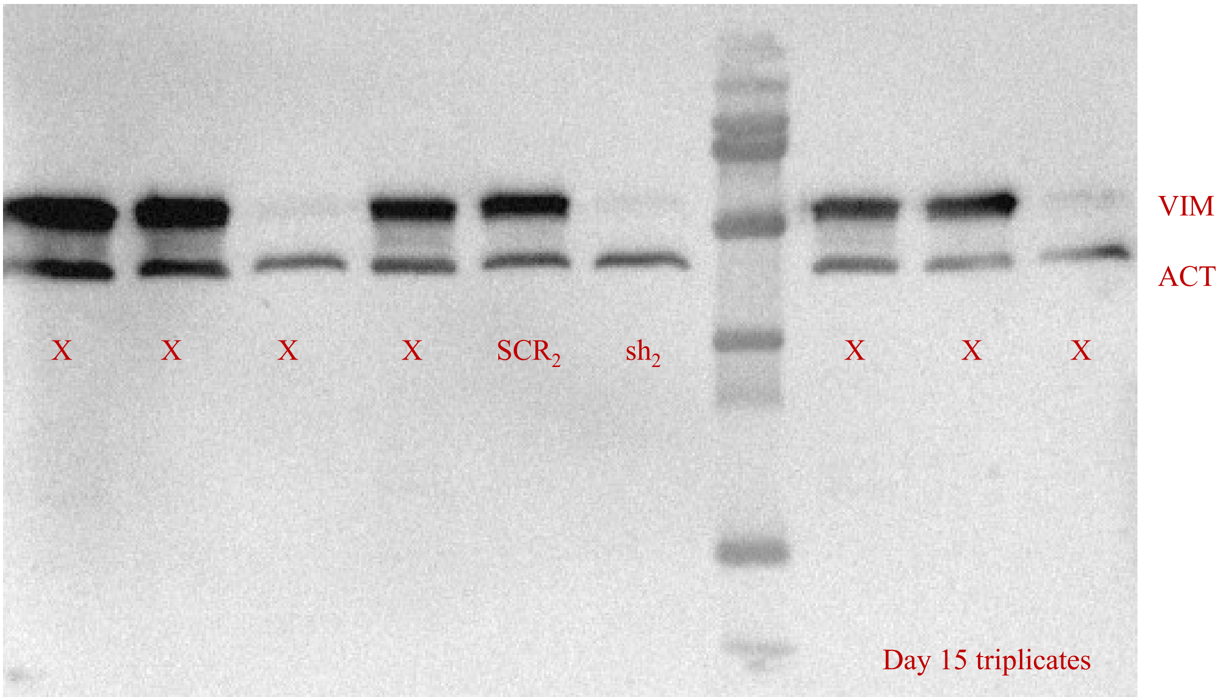

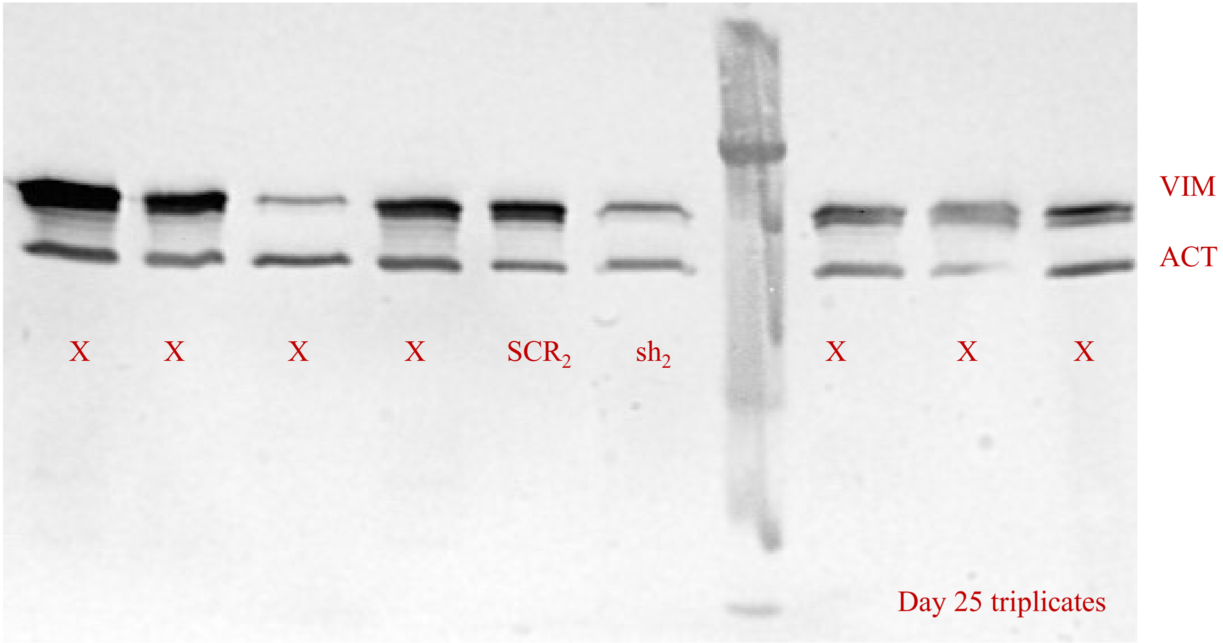
VIM
ACT
X
X
X
X
SCR_2
sh_2
X
X
X
Day 25 triplicates

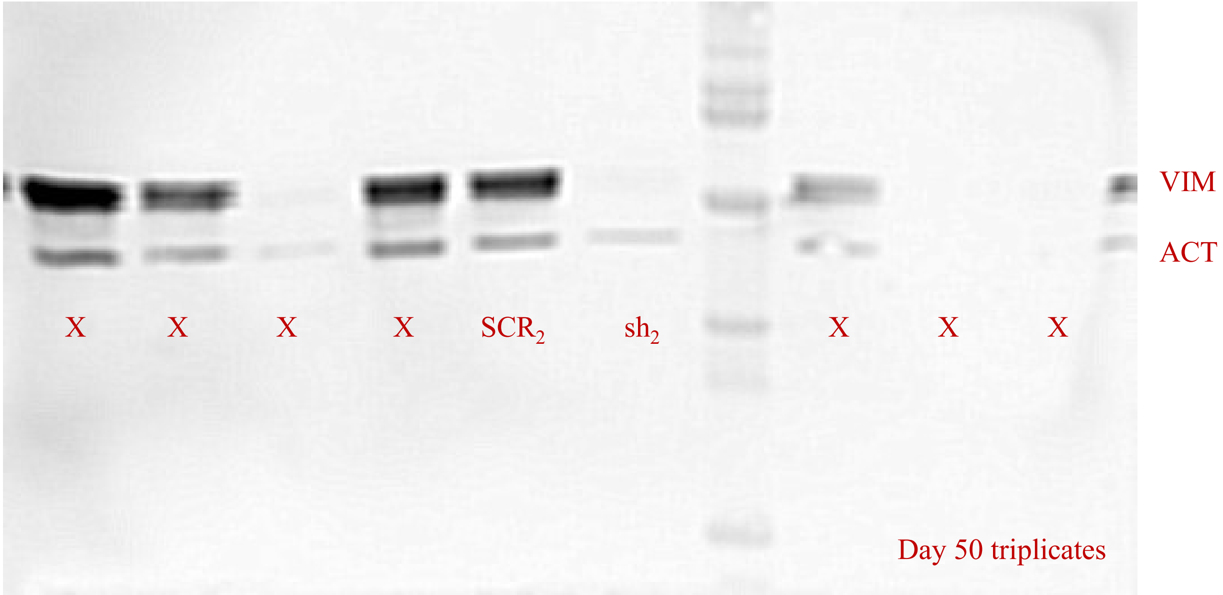
VIM
ACT
X
X
X
X
SCR_2
sh_2
X
X
X
Day 50 triplicates

Detection of TPI and MAT2A for N, SCR and sh of HD1 in triplicates

- Aim: Comparison of protein expression of TPI and MAT2A in sh$_1$ and SCR$_1$ at each detection day separately
- Same samples were inserted identically to VIM gels (20µg) and detected in the same run

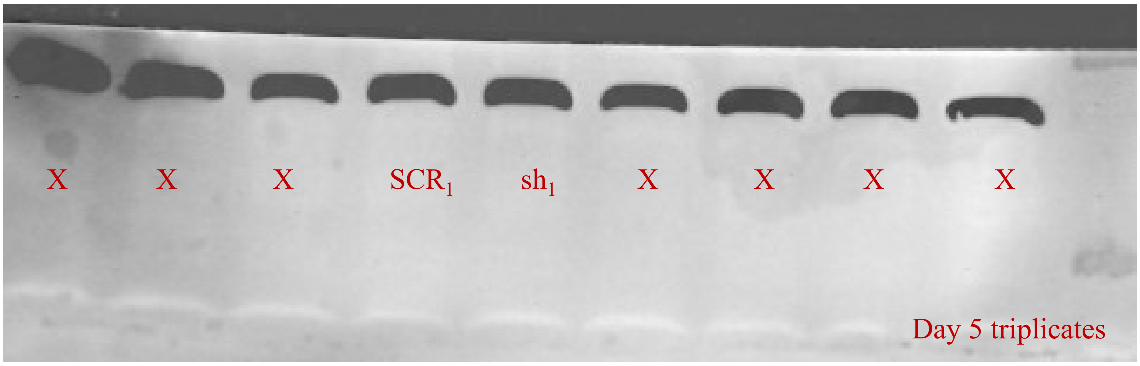

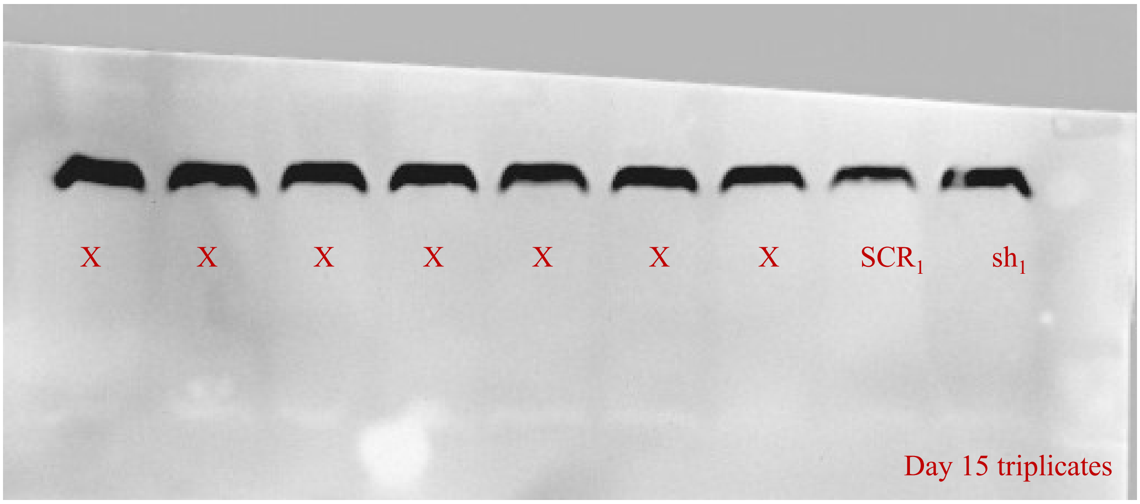

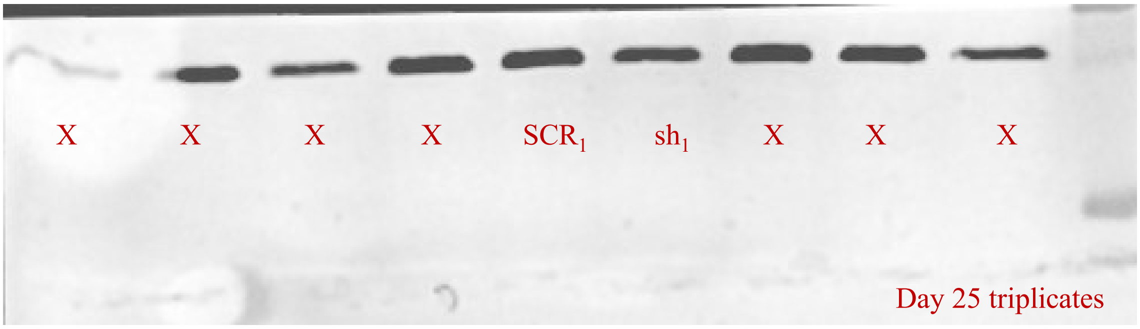

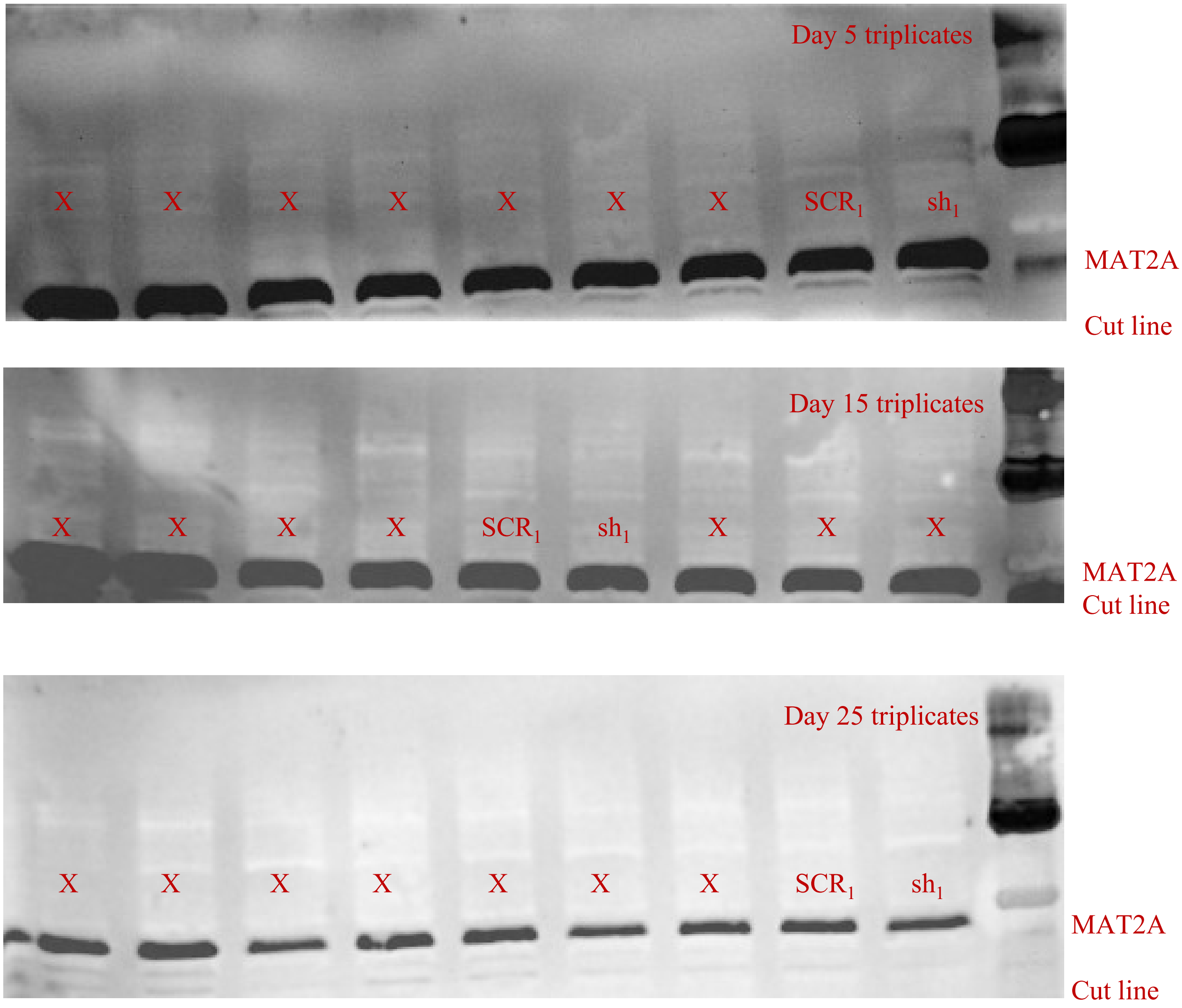
Day 5 triplicates
X X X X X X X SCR1 sh1
MAT2A
Cut line
Day 15 triplicates
X X X X SCR1 sh1 X X X
MAT2A
Cut line
Day 25 triplicates
X X X X X X X SCR1 sh1
MAT2A
Cut line

Detection of TPI and MAT2A for N, SCR and sh of HD2 in triplicates

- Aim: Comparison of protein expression of TPI and MAT2A in sh_2 and SCR_2 at each detection day separately
- Same samples were inserted identically to VIM gels (20µg) and detected in the same run

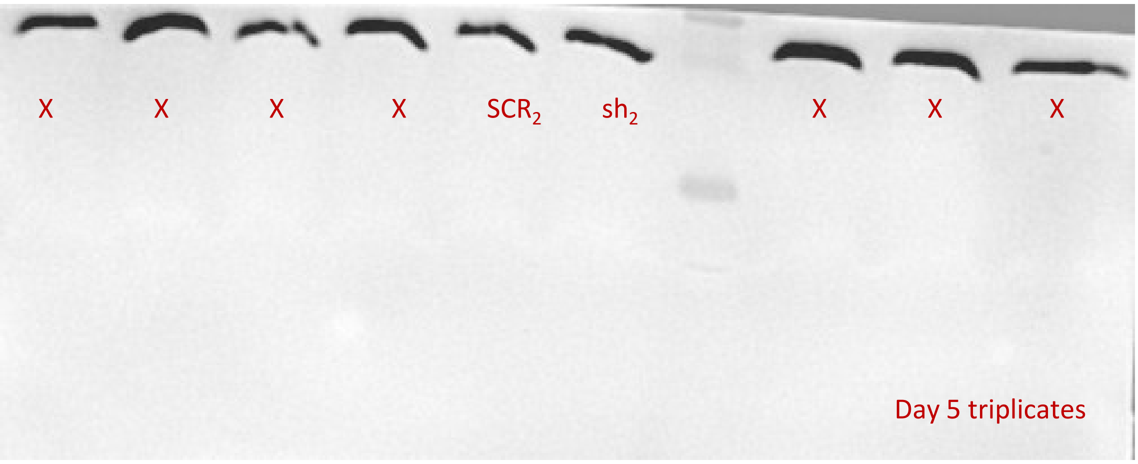

Cut line
TPI

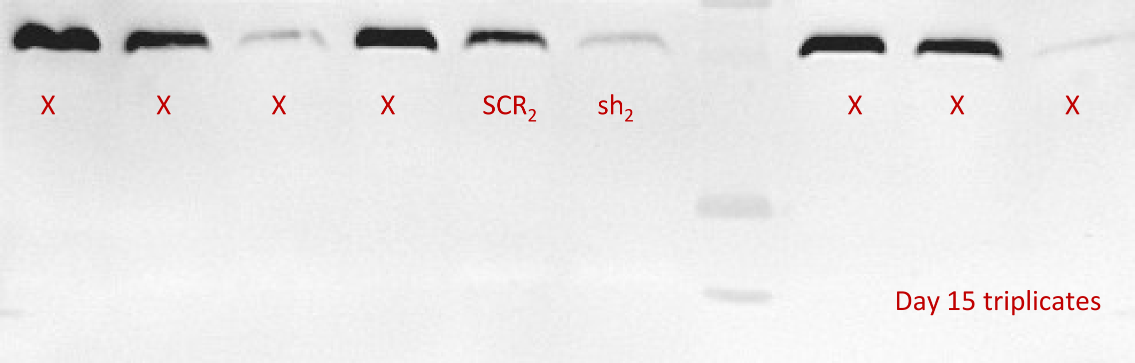

Cut line
TPI

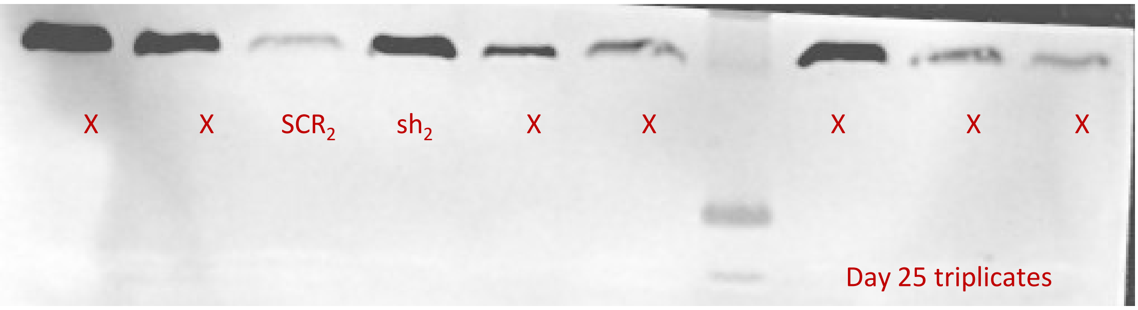

Cut line
TPI

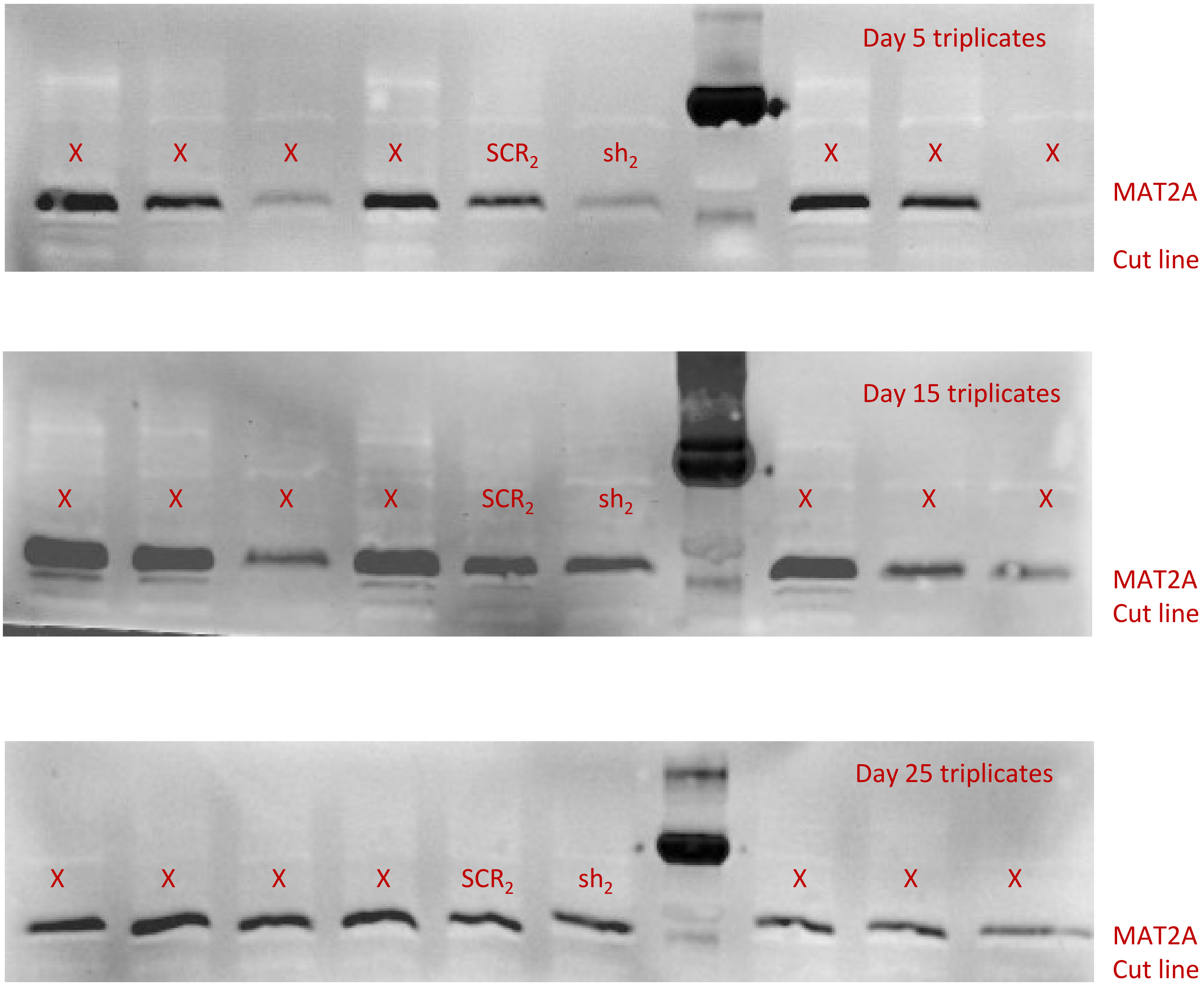
Day 5 triplicates
X X X X SCR2 sh2 X X X
MAT2A
Cut line
Day 15 triplicates
X X X X SCR2 sh2 X X X
MAT2A
Cut line
Day 25 triplicates
X X X X SCR2 sh2 X X X
MAT2A
Cut line
